# Nitrate and ammonium, the yin and yang of nitrogen uptake: a time-course transcriptomic study in rice

**DOI:** 10.3389/fpls.2024.1343073

**Published:** 2024-08-23

**Authors:** Pierre-Mathieu Pélissier, Boris Parizot, Letian Jia, Alexa De Knijf, Vera Goossens, Pascal Gantet, Antony Champion, Dominique Audenaert, Wei Xuan, Tom Beeckman, Hans Motte

**Affiliations:** ^1^ Department of Plant Biotechnology and Bioinformatics, Ghent University, Ghent, Belgium; ^2^ VIB Center for Plant Systems Biology, Ghent, Belgium; ^3^ State Key Laboratory of Crop Genetics & Germplasm Enhancement and MOA Key Laboratory of Plant Nutrition and Fertilization in Lower Middle Reaches of the Yangtze River, Nanjing Agricultural University, Nanjing, China; ^4^ Center for Bioassay Development and Screening (C-BIOS), Ghent University, Ghent, Belgium; ^5^ VIB Screening Core, Ghent, Belgium; ^6^ UMR DIADE, Université de Montpellier, IRD, CIRAD, Montpellier, France

**Keywords:** transcriptome, rice, co-expression network, nitrogen, OsRLI1, OsEIL1

## Abstract

Nitrogen is an essential nutrient for plants and a major determinant of plant growth and crop yield. Plants acquire nitrogen mainly in the form of nitrate and ammonium. Both nitrogen sources affect plant responses and signaling pathways in a different way, but these signaling pathways interact, complicating the study of nitrogen responses. Extensive transcriptome analyses and the construction of gene regulatory networks, mainly in response to nitrate, have significantly advanced our understanding of nitrogen signaling and responses in model plants and crops. In this study, we aimed to generate a more comprehensive gene regulatory network for the major crop, rice, by incorporating the interactions between ammonium and nitrate. To achieve this, we assessed transcriptome changes in rice roots and shoots over an extensive time course under single or combined applications of the two nitrogen sources. This dataset enabled us to construct a holistic co-expression network and identify potential key regulators of nitrogen responses. Next to known transcription factors, we identified multiple new candidates, including the transcription factors OsRLI and OsEIL1, which we demonstrated to induce the primary nitrate-responsive genes *OsNRT1.1b* and *OsNIR1*. Our network thus serves as a valuable resource to obtain novel insights in nitrogen signaling.

## Introduction

Nitrogen, mainly in the form of nitrate (NO_3_^-^) or ammonium (NH_4_^+^), is a key nutrient for plant development and a limiting factor for crop yield and grain quality (Makino, 2011). Nitrogen application soared with the green revolution and is expected to keep growing (Good et al., 2004; Food and Agriculture Organization of the United Nations [FAO], 2017). However, major staple crops use less than half of the nitrogen applied through fertilizers, the rest being lost by leaching or volatilization, causing economic losses and ecological damages such as eutrophication and greenhouse gas emissions (Raun and Johnson, 1999; Bouwman et al., 2002; Robertson and Vitousek, 2009; Sutton et al., 2011; Coskun et al., 2017; Beeckman et al., 2018, Beeckman et al., 2024). Therefore, a better understanding of how plants respond and assimilate nitrogen is of great interest to improve their nitrogen use efficiency (NUE). Attempts to improve NUE have often targeted single genes involved in nitrogen metabolism or transport (McAllister et al., 2012). In contrast, transcription-factor-centered approaches yielded promising results, as one transcription factor can potentially regulate several genes Past research has elucidated complex nitrogen-related pathways governed by transcription factors. However, further exploration is warranted to advance our understanding of regulatory networks involved in NUE, particularly in crops (Ueda and Yanagisawa, 2018).

NUE is a complex trait not only because of complex signaling, but also because plants react differently to nitrate and ammonium. Most plants prefer nitrate over ammonium and are stressed when ammonium is provided alone or in high quantities (Kronzucker et al., 2001; Britto and Kronzucker, 2013; Bittsanszky et al., 2015; Hachiya and Sakakibara, 2016), but rice is tolerating ammonium reasonably well (Sasakawa and Yamamoto, 1978). Besides fulfilling its role as a nutrient, nitrate also acts as a signaling molecule at the local and the systemic level (Crawford, 1995; Krouk et al., 2010; Xuan et al., 2017; Pélissier et al., 2021), inducing responses in Arabidopsis as early as 3 minutes after treatment (Krouk et al., 2010) while this appears to not be the case for ammonium. At least in Arabidopsis, and to some extent in rice, knowledge on nitrate response regulation increased considerably due to systems biology approaches aiming at characterizing transcriptional networks (Gaudinier et al., 2018; Varala et al., 2018; Ueda et al., 2020). Both in rice and Arabidopsis, nitrate binds to NITRATE TRANSPORTER (NRT) transceptors (OsNRT1.1b or AtNRT1.1 in rice or Arabidopsis, respectively), which trigger Ca^2+^ signaling and activate different Ca^2+-^sensor protein kinases (CPKs) that phosphorylate NIN-LIKE PROTEIN (NLP) transcription factors: AtNLP6 and AtNLP7 in Arabidopsis or OsNLP3 in rice. As a result, NLPs are retained in the nucleus and regulate hundreds of nitrate responsive genes triggering a complex cascade of systemic signaling and feedback loops (Marchive et al., 2013; Guan et al., 2017; Liu et al., 2017; Hu et al., 2019; Alvarez et al., 2020). Nitrate is also perceived directly by AtNLP7, which leads to a de-repression of this transcription factor (Liu et al., 2022).

In contrast to nitrate, no ammonium signaling mechanism has been discovered, at least not in plants. Ammonium-induced changes in the root system architecture or other responses seemed to be primarily caused by changes in internal cellular pH and auxin mobility rather than changes induced by a biochemical signaling pathway (Jia et al., 2020; Meier et al., 2020; Hachiya et al., 2021). These results argue that ammonium, in contrast to nitrate, does not directly affect a transcriptional pathway. Notably, nitrate and nitrate signaling affect ammonium responses and NRT1.1-dependent signaling plays crucial roles in controlling ammonium uptake and assimilation (Jian et al., 2018; Wu et al., 2019; Fang et al., 2021; Yan et al., 2023), while nitrate is reduced to ammonium during assimilation and partially elicits an ammonium response (Wang et al., 2004). Conversely ammonium affect nitrate uptakes and other responses (Wang et al., 2009b; Hachiya and Sakakibara, 2016). Hence, there is a clear interaction between these two nitrogen sources and variations in one will inevitably affect the overall response. This interplay is important to consider in network analysis, and could help to uncover regulatory mechanisms that might be overlooked if only one nitrogen source is considered. Genes that respond to both nitrogen sources, for example, can complicate the identification of specific responses to one nitrogen source. Considering both allows for distinguishing between the different responses, can refine network analysis and is potentially instrumental in elucidating otherwise overlooked regulatory mechanisms. Although several studies investigated the responses to nitrate, ammonium and their co-application in Arabidopsis (Patterson et al., 2010; Ristova et al., 2016) and rice (Obertello et al., 2015; Chandran et al., 2016; Yang et al., 2017; Fu et al., 2023), they often lack an extensive time-course necessary for construction of gene regulatory networks.

Here, to enable a better view on the nitrogen response and its regulatory network in rice, we conducted an extensive time-course and genome-wide transcriptional analysis both in roots and shoots and in responses to ammonium, nitrate, or the combination of both. We used this dataset to construct a gene co-expression network which allowed us to reveal several transcription factors with a possible role in nitrogen signaling, and showed that the transcription factors OsRLI1 and OsEIL1 are sufficient to activate a nitrate response. As such, our dataset does not only provide a new resource to retrieve the genome-wide gene expression in response to different nitrogen sources, but is also valuable to get insights into nitrogen signaling in rice, and by extension, in crops.

## Results

### Phenotypic responses of rice to different nitrogen forms

**Figure 1:**
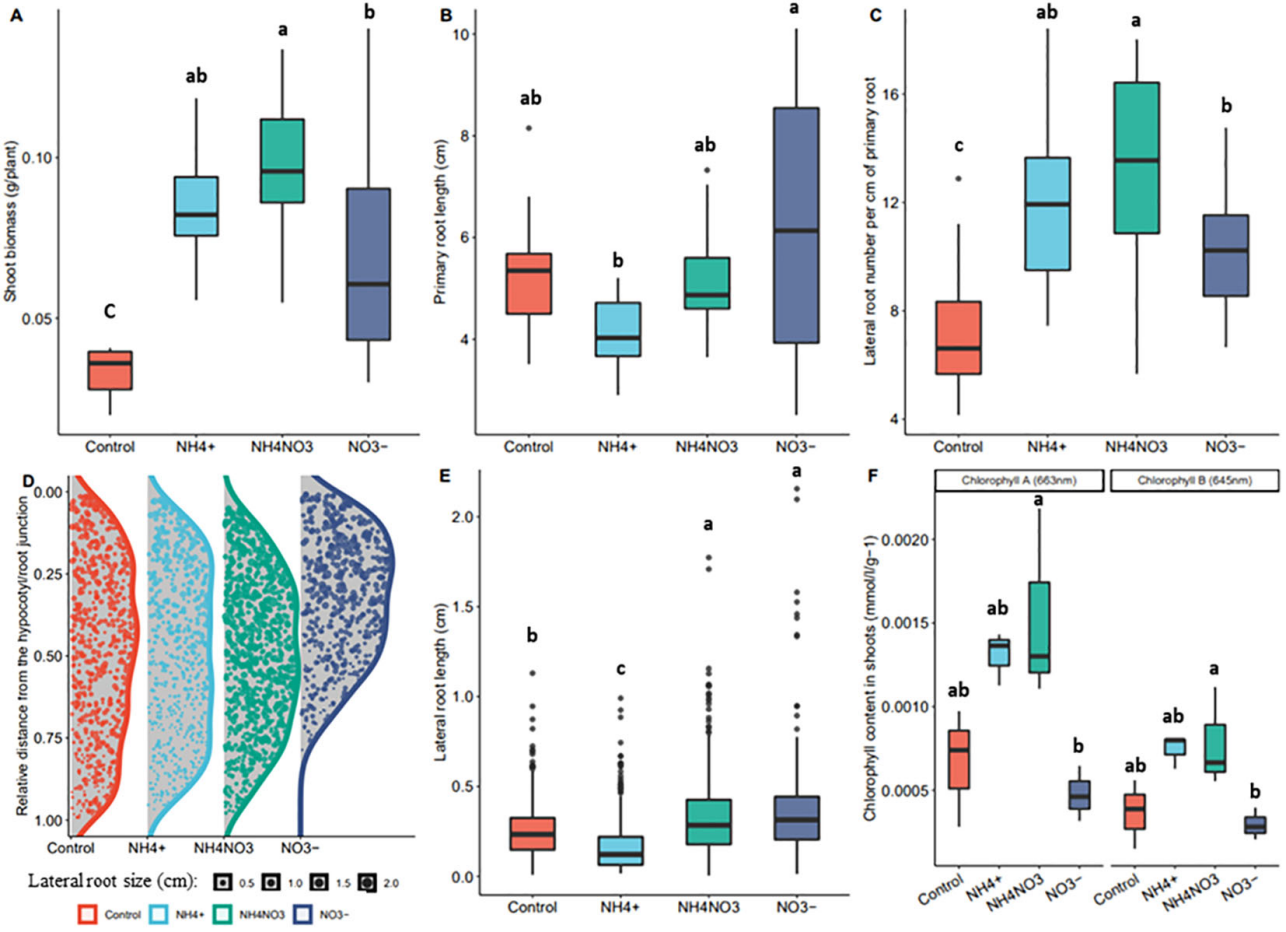
Rice phenotype in response to different nitrogen forms. Effects of nitrate (NO_3_^-^), ammonium (NH_4_^+^) and equimolar combination of both forms (NH_4_NO_3_) on rice seedlings grown for 5 days on nitrogen free medium and supplemented with the different treatments for 10 days. Boxplots lower side, middle line and upper side represent the median, the 25^th^ and 75^th^ percentiles, respectively (interquartile range or IQR). Boxplots whiskers represent data falling within a 1.5xIQR distance, measurements beyond this distance are plotted as single points. **(A)** fresh shoot biomass per plant (n=15). **(B)** Primary root length (n=15) **(C)** Emerged lateral root density (n=15) **(D)** Density plot of the distribution of lateral roots over the primary root. On the Y axis, 0.00 represents the root-hypocotyl junction, and 1.00 represents the root tip. The data is normalized on the primary root length. The length of each lateral root is represented by the size of the dots. **(E)** Average lateral root length (n=15) **(F)** Leaf blade chlorophyll content (samples (n) are 5 seedlings pooled together, n=3). Different letters correspond to the post-hoc Tuckey’s test significance (p.value=0.05), performed after an ANOVA test, and showing significant differences between the samples.

To investigate the response of rice to different nitrogen forms, we used a hydroponic system in which ammonium and/or nitrate could be supplemented to the medium. 5mM of nitrate (NO_3_^-^ as KNO_3_), 5mM of ammonium (NH_4_^+^ as (NH_4_)_2_SO_4_), an equimolar combination of both nitrogen forms (2.5mM of NH_4_NO_3_) or a control (5mM K^+^ as K_2_SO_4_) with potassium (K^+^) balanced at 5mM among all treatments as K_2_SO_4_, were supplemented into the nitrogen-free growing media of the rice seedlings 5 days after germination and the seedlings were let grown for 10 more days before phenotyping (see Materials & Methods for details on the procedure). In our set-up, supplementation with ammonium and nitrate had a similar positive effect on shoot biomass, while co-application of both forms showed a synergistic positive effect (**Figure 1A**). The lateral root density positively correlated with the shoot biomass and showed a similar synergistic response to the combined treatment. The root system treated with nitrate had a long primary root with long lateral roots close to the root-hypocotyl junction, while the ammonium-supplemented root system had a dense network of small lateral roots evenly spread over the primary root (**Figures 1B–E**). Co-application seemed to result in a combination of the two phenotypes. Finally, we observed an increase in leaf chlorophyll content upon treatment by ammonium or ammonium-nitrate but not by nitrate alone (**Figure 1F**).

### Dynamic rice nitrogen transcriptome

**Figure 2:**
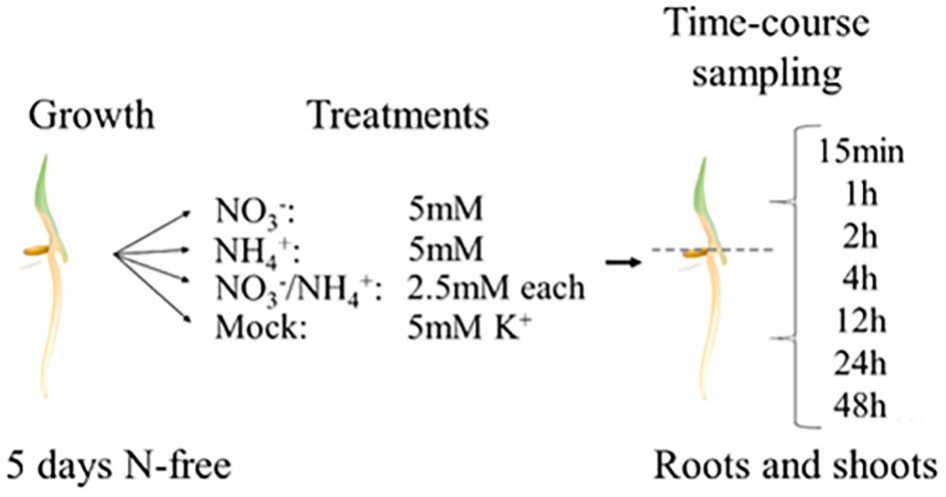
Experimental set-up of the RNA-seq.

We used the same hydroponic system as described above to collect samples for the transcriptomic analysis, but rice tissues were harvested soon after the nitrogen supplementation (see Material and Methods for details). In Arabidopsis, early response genes are induced as early as 12 minutes (*NITRITE REDUCTASE1 (NIR1*)), 15 minutes (*NRT2.1* and *NITRATE REDUCTASE1* (*NIA1*)) or 20 minutes (*NITRATE TRANSPORTER1.1 (NRT1.1)*) after nitrate treatment (Krouk et al., 2010). Therefore, to capture relevant transcriptional profiles, we sampled root and shoot tissue separately immediately (0h), 15 minutes, 1h, 2h, 4h, 12h, 24h and 48h after treatment and used these samples for RNA sequencing (RNA-seq) thereby generating an extensive dataset covering the nitrogen transcriptional responses in rice (**Figure 2**).

We performed a pair-wise differential analysis to assess differential expression for each time point and treatment (**Supplementary Dataset S1**, **Supplementary Dataset S2**). Considering an absolute fold-change >2, and an adjusted p.value (FDR) < 0.05, a significant number of genes were differentially expressed by the treatments in the shoot or root and over the time-course (**Supplementary Figures S1**–**S3**; **Supplementary Dataset S1**, **Supplementary Dataset S2**). Nitrate, alone or in combination with ammonium, rapidly induced over 250 genes in the roots within 15 minutes (**Supplementary Figures S1**). This list includes homologues of Arabidopsis primary nitrate response genes such as *LOB DOMAIN-CONTAINING PROTEIN37/38/39 (LBD37/38/39), NITRATE-INDUCIBLE GARP-TYPE TRANSCRIPTIONAL REPRESSOR1 (NIGT1), NRT1.1*, nitrate and nitrite reductases*, GLUCOSE-6-PHOSPHATE DEHYDROGENASE3 (G6PDH3)* and *ARABIDOPSIS NAC DOMAIN-CONTAINING protein 4 (NAC4)* (**Supplementary Dataset S1**). In contrast, the response to ammonium was very weak at the 15 minutes-timepoint but a high number of differentially expressed genes was observed after 1h (**Supplementary Figures S1**, **S3**; **Supplementary Dataset S1**), including the transporter-encoding *AMMONIUM TRANSPORTER1.2* (*OsAMT1.2*) and *OsAMT2.2* or the amino acid assimilation enzyme-encoding *ALANINE AMINOTRANSFERASE1* (*OsAlaAT1*), *OsAlaAT2*, *ASPARAGINE SYNTHETASE1* (*OsASN1*), *PHOSPHOENOL PYRUVATE CARBOXYKINASE1* (*OsPPCK1*), and *GLUTAMATESYNTHASE1* (*OsGLT1*). The highest number of differentially expressed genes was in general observed with the combined treatment of ammonium and nitrate. The majority of these genes were also affected by either the ammonium or nitrate treatment (**Supplementary Figure S1**). Hence, the combined ammonium-nitrate response seems to largely reflect the sum of the individual responses.

In the shoot, a strong response only occurred from 4h onwards, primarily attributable to the nitrate treatment. The ammonium treatment resulted in a slower response, but from 12h onwards, large transcriptomic changes were observed as well (**Supplementary Dataset S2**, **Supplementary Figure S2**).

### Co-expression network analysis identifies unique gene clusters responsive to nitrate and ammonium treatments in roots and shoots

To analyze the gene response profiles towards the different treatments, we built a co-expression network using the R package WGCNA (Langfelder and Horvath, 2008) for the most varying genes in the roots (18457) and shoots (18343). The network revealed 54 co-expression clusters in the roots and 55 in the shoots (**Supplementary Figures S4**, **S5**; **Supplementary Datasets S1**, **S2**). The accompanying edge and node tables, compatible with network visualization tools such as Cytoscape or Gephi can be downloaded at https://osf.io/2uzd3/. To provide access to these resources, we generated a Shiny app **Supplementary Figure S6**), https://www.psb.ugent.be/shiny/rice-response-to-nitrogen/). The user can query any of the 42189 rice genes to display the expression profile in response to the different nitrogen treatments. If the gene is also included in the 18457 genes or 18343 genes used for the co-expression network, the Eigengene of its WGNCA cluster and a correlation coefficient with highly correlated genes (biweight midcorrelation, computed during the gene co-expression network creation) is also displayed. The latter is also shown in **Supplementary Datasets S3** and **Supplementary Dataset S4**, which facilitate the identification of highly co-expressed gene pairs. The cluster membership and associated p values indicating the contribution to the cluster profile for each gene as well as the number of connections to other genes within the same cluster are indicated in **Supplementary Datasets S1**, **S2.**

**Figure 3:**
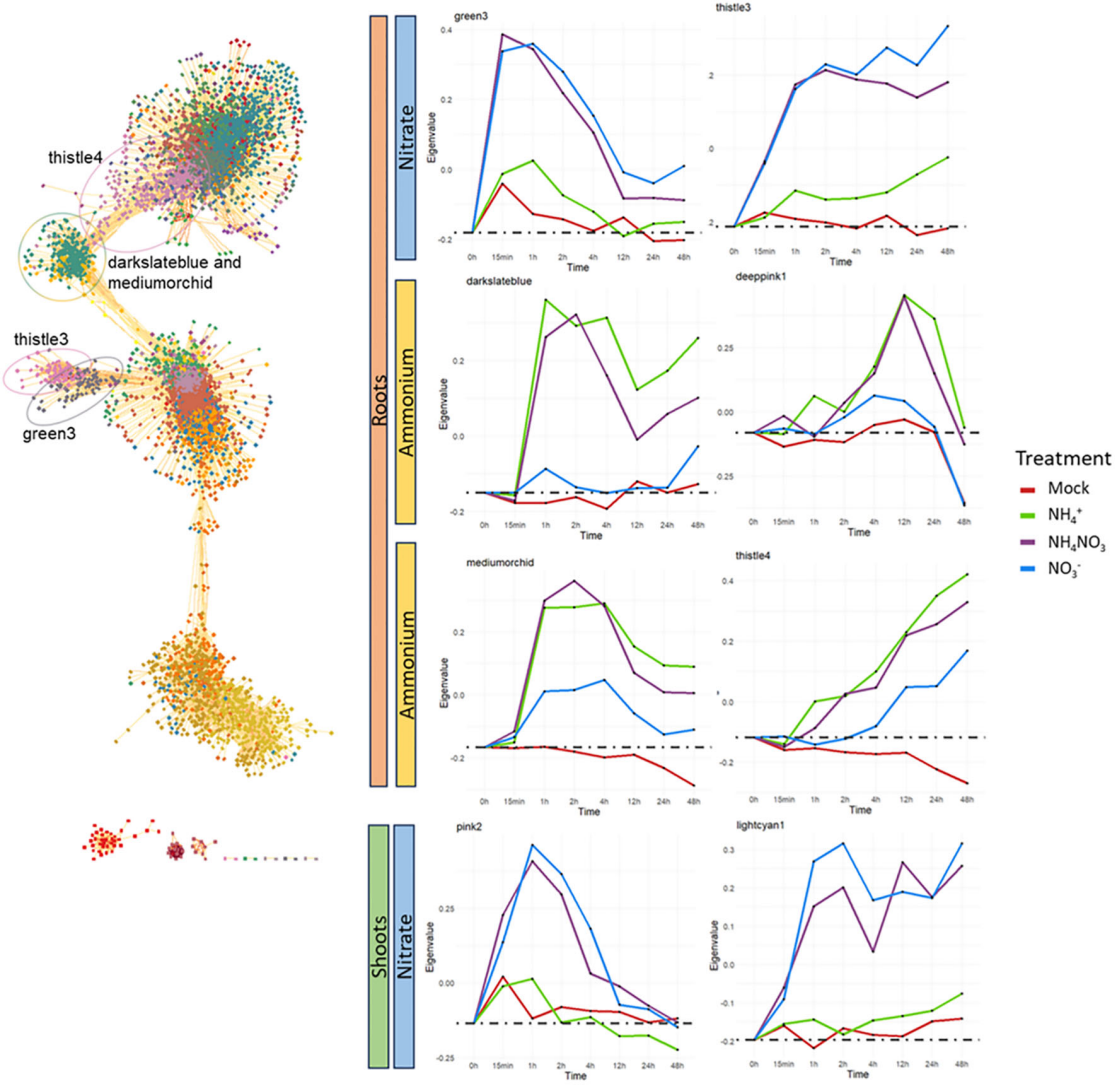
WGNCA co-expression network in the root and examples of WGNCA clusters in roots and shoots with a specific response to nitrate or ammonium. The expression profile of the clusters is shown by the eigengene, a representative for the overall expression and calculated as the first principal component of the gene expression data in the respective cluster. Root-specific clusters are indicated in the network. The cluster deeppink1, which is a relative small cluster, is not indicated.

In the roots, we identified clusters specifically and early induced by nitrate (nitrate and ammonium-nitrate treatments only) containing transiently (‘green3’) or constitutively induced genes (‘thistle3’) (**Figure 3**; **Supplementary Figure S4**). We identified two clusters specifically induced by ammonium (‘darkslateblue’ and ‘deeppink1’). Two clusters of genes were induced by ammonium and with an approximately 4h delay by nitrate or weaker induction by nitrate, possibly due to the nitrate to ammonium reduction (‘mediumorchid’, ‘thistle4’). We identified small clusters with a specific response to ammonium (‘yellow3’) or nitrate (‘indianred3’), but no or very weak response to the combination of the two nitrogen forms, indicative for a countereffect of the other nitrogen form on these genes. Vice versa, we did not identify clusters of genes induced by the ammonium-nitrate treatment only. Some other clusters show a similar response to all nitrogen forms, and are likely related to the nitrogen nutrition. Most other clusters showed a high response in the mock as well or show irregular or variable expression profiles (**Supplementary Figure S4**).

In the shoots, we identified early responsive and nitrate-specific clusters that are similar to the nitrate-specific clusters in the roots, including a transient (‘pink2’, similar to ‘green3’) and a constitutive cluster of upregulated genes (‘lightcyan1’, similar to ‘thistle3’). Also a cluster of genes exclusively induced by nitrate could be observed (‘plum4’), similar as the ‘indianred3’ cluster in the roots. Contrary to the roots, we did not identify an ammonium-specific cluster in the shoots. Moreover, many more shoot clusters exhibit irregular patterns or show similar responses in the mock as in the treatments, making them of less interest. Overall, our co-expression networks revealed clusters of genes illustrating strong temporal and differential biological responses to the different forms of nitrogen provided.

To further investigate the clusters nature, we conducted a gene-ontology enrichment analysis (**Supplementary Datasets S5**, **S6**). We first compared the nitrate-specific clusters in the roots (‘green3’, ‘thistle3’) and the shoots (‘pink2’, ‘lightcyan1’). The genes ontologies enriched in both roots and shoots nitrate-specific clusters are highly similar and many genes are retrieved in both clusters: 72.8% of the 125 genes composing the nitrate-specific shoot clusters are retrieved in the 414 genes composing the nitrate-specific root clusters. The genes present in all these clusters are primarily related to nitrate assimilation and nitrate transport.

Highly enriched terms for the ammonium-specific clusters in the roots ‘darkslateblue’ and ‘deeppink1’ are mainly related to ammonium or amino acid assimilation and cellular respiration or ATP production. The ‘indianred3’ root cluster and the ‘plum4’ shoot cluster, containing genes that are exclusively induced by nitrate alone, are both highly enriched in iron-related terms. The root cluster ‘yellow3’ showing an exclusive response to ammonium alone, mainly concerns genes related to oxidative stress (**Supplementary Datasets S3**, **S4**).

### Nitrogen network highlight known and novel transcription factors involved in the nitrate specific response

**Figure 4:**
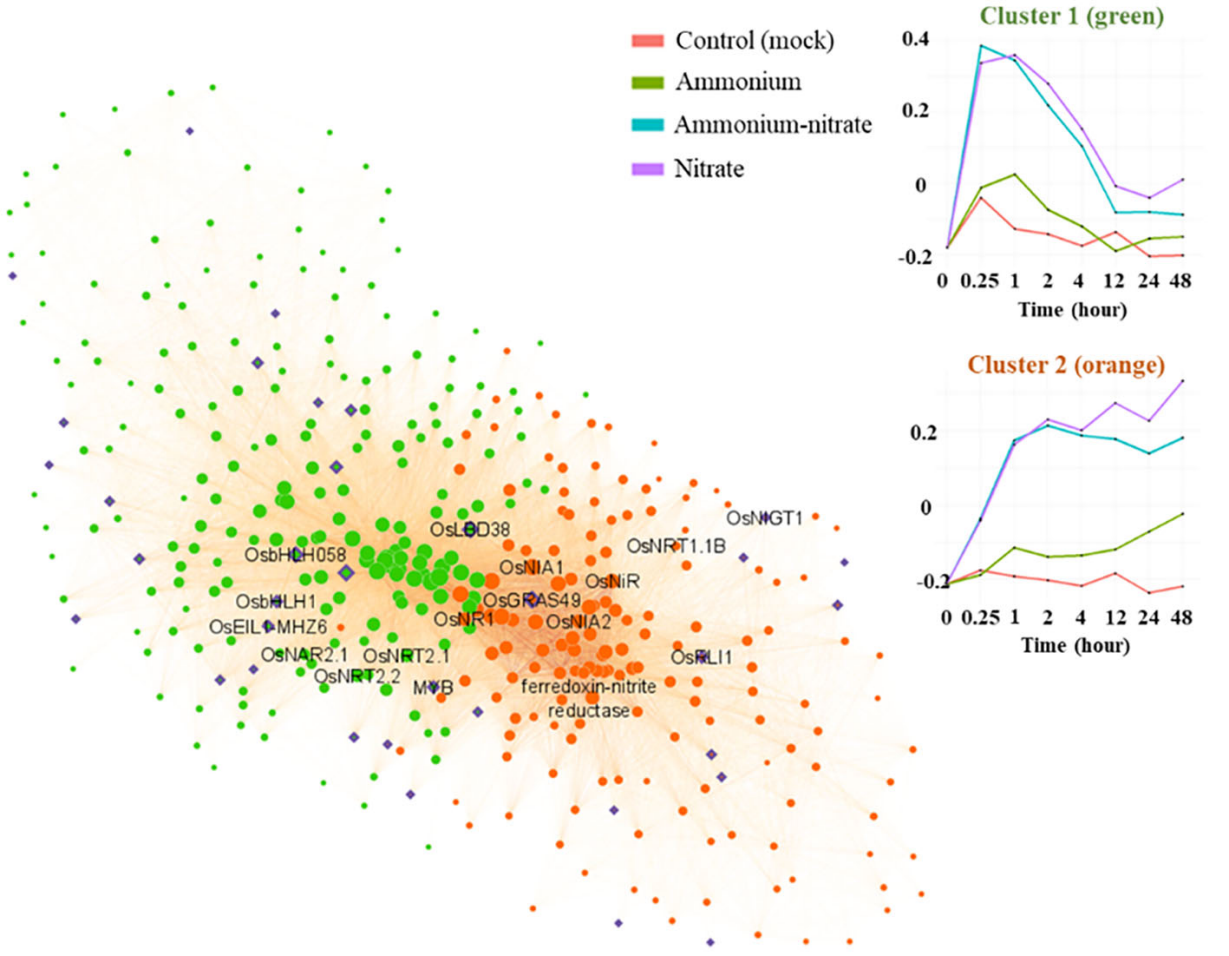
Co-expression network for the two main nitrate specific clusters. The green genes belong to the cluster ‘green3”, the orange ones to ‘thistle3’. The purple genes correspond to transcription factors. The transcription factors present in these clusters, together with their number of connections to other genes, are available in **Table 1**.

For further analysis of the co-expression network, we zoomed in on the two main nitrate-specific clusters in the root network (‘green3’ and ‘thistle3’) containing genes that were rapidly induced upon nitrate (**Figure 4**; **Supplementary Table S1**). With for example *OsNRT1.1B* (Os10g40600) and *NITRATE REDUCTASE1* (*OsNR1*) (Os08g36480), this group contains typical nitrate sentinel genes. In the same group, we identified 38 transcription factors based on PlantTFDB v5.0 (https://planttfdb.gao-lab.org/) (Tian et al., 2019) (**Supplementary Dataset S1**). Several of these transcription factors have a high module membership and a high number of connections within one of the two clusters and could be designated as ‘hub’ genes with potentially an important role in the nitrate response or signaling (**Figure 4**, **Table 1**). A highly connected transcription factor in ‘green3’ is *OsLBD38* (Os03g41330) which homologues were shown to be involved in nitrogen signaling in Arabidopsis or other species (Rubin et al., 2009; Teng et al., 2022), while *OsLBD38* seems to be part of a conserved regulatory cluster between Arabidopsis and rice (Obertello et al., 2015). OsLBD38 is also the most connected transcription factor in the shoot cluster ‘lightcyan1’ (**Supplementary Dataset S2**). *OsNIGT1* (Os02g22020), known to be an important transcriptional regulator of the nitrate signaling as well, is also present in ‘thistle3’ (**Figure 4**, **Table 1**; **Supplementary Dataset S1**) (Maeda et al., 2018). Several transcription factors have come forward that have not been previously related to nitrate response. *OsGRAS49* (Os11g47890) for instance, which is to our knowledge not reported to have a role in the nitrate response, is a potential ‘hub’ transcription factor in the nitrate specific clusters in both roots and shoots (**Supplementary Dataset S1**, **S2**).

**Table 1:** Transcription factors with at least one connection in the clusters green3 or thistle3 as presented in **Figure 4**.

Cluster	LOCUS ID	Gene name	Transcription factor family (PlantTFDB v5.0)	MM WGNCA cluster	p.MM WGNCA cluster	number of connections
**Cluster 1 (green3)**	LOC_Os05g38140.1	OsbHLH058	bHLH	0.949189	4.43E-15	154
	LOC_Os03g62230.1		C2H2	0.930715	2.61E-13	144
	LOC_Os03g41330.1	OsLBD38	LBD	0.839871	1.22E-08	112
	LOC_Os07g43530.1	OsbHLH1	bHLH	0.924495	8.03E-13	106
	LOC_Os11g06010.1	OsbHLH151	bHLH	0.924182	8.48E-13	105
	LOC_Os05g37730.1		MYB	0.869067	9.67E-10	92
	LOC_Os03g20790.1	OsEIL1	EIL	0.853357	4.05E-09	91
	LOC_Os08g43090.1	OsbZIP68	bZIP	0.805793	1.33E-07	81
	LOC_Os05g45020.1	OsC3H37	C3H	0.795072	2.57E-07	76
	LOC_Os01g04930.1		MYB	0.769732	1.05E-06	67
	LOC_Os01g43550.2	OsWRKY12	WRKY	0.836758	1.55E-08	58
	LOC_Os06g05890.1	OsBBX16	DBB	0.78879	3.71E-07	57
	LOC_Os09g31400.1	OsEIL3	EIL	0.791398	3.19E-07	56
	LOC_Os03g20780.1	OsEIN3	EIL	0.771365	9.68E-07	51
	LOC_Os12g21700.1	OsC3H66	C3H	0.778115	6.75E-07	44
	LOC_Os03g50920.1	OsZHD11	ZF-HD	0.860267	2.2E-09	42
	LOC_Os01g43590.2	OsHsfC1a	HSF	0.797629	2.21E-07	38
	LOC_Os03g13400.1	OsIDD14	C2H2	0.804705	1.43E-07	37
	LOC_Os08g38220.1	OsDof24	Dof	0.823533	4.09E-08	24
	LOC_Os04g32590.1		Trihelix	0.718961	1.11E-05	23
	LOC_Os01g45090.1	OsMYB8	MYB	0.777949	6.81E-07	7
	LOC_Os02g52670.1	OsDERF5	ERF	0.639761	0.000186	2
**Cluster 2 (thistle3)**	LOC_Os11g47890.1	OsGRAS49	GRAS	0.942288	2.37E-14	112
	LOC_Os04g56990.1	OsRLI1	G2-like	0.937522	6.73E-14	107
	LOC_Os09g21180.1	OsHox25	HD-ZIP	0.815732	6.99E-08	64
	LOC_Os10g18099.1		WRKY	0.865895	1.31E-09	60
	LOC_Os02g22020.1	OsNIGT1	G2-like	0.881506	2.71E-10	59
	LOC_Os01g64020.1	OsbZIP11	bZIP	0.905257	1.53E-11	46
	LOC_Os03g46790.1	OsbHLH022	bHLH	0.841582	1.07E-08	38
	LOC_Os02g06910.1	OsARF6a	ARF	0.831721	2.27E-08	27
	LOC_Os07g25710.3	OsPHR2	G2-like	0.714212	1.35E-05	21
	LOC_Os07g02800.2		G2-like	0.705864	1.89E-05	16
	LOC_Os11g47870.1		GRAS	0.816776	6.51E-08	8
	LOC_Os03g52450.1	OsTIFY1b	GATA	0.76735	1.19E-06	7
	LOC_Os12g06640.1		Trihelix	0.656455	0.00011	3

The module membership (MM) and the associated p.value (p.MM) indicates how strongly a gene is associated with the cluster and is calculated based on the gene’s connectivity within the cluster, reflecting its contribution to the overall. The number of connections shows the number of other genes within the same WGNCA cluster that show a co-expression coefficient of at least 0.1 with the gene.

#### OsEIL1 and OsRLI1 affect the expression of core nitrate responsive genes

**Figure 5:**
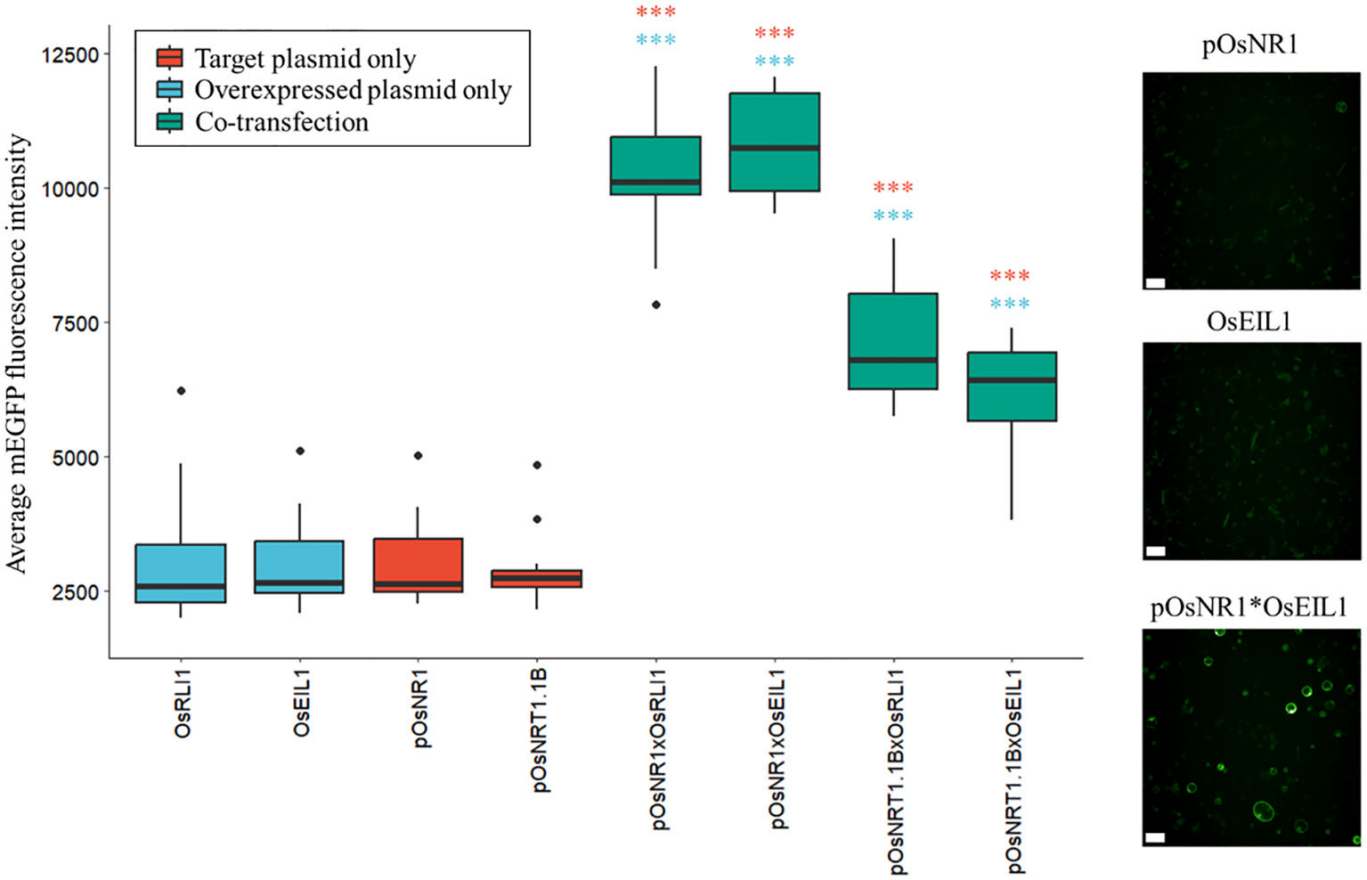
Protoplast transactivation assay. Induction of nitrate response genes by the two selected transcription factors in a rice protoplast transactivation assay. The boxplots show the average mEGFP fluorescence intensity per transfected protoplast (min. 118 protoplasts per condition, average 408) in one well (n=16). Samples (green) are co-transfected with the indicated combinations of inducer and target plasmids. The negative controls are only transfected with the inducer plasmid (blue) or with the target plasmids (red). Significance was determined by a one-way ANOVA followed by a Tukey’s post-hoc test (***p < 1.10^-6^, blue: sample versus the transcription factor control, red: versus the promoter of the reporter control). Confocal images show negative controls (pOsNR1 and pOsEIL1) and activation of OsNR1 by OsEIL1 (pOsNR1*OsEIL1) in the mEGFP channel (emission: 522nm, excitation: 488nm). Scale bars: 50µm.

To assess these transcription factors possible role in nitrate signaling, we selected the top hub transcription factors in ‘green3’ and ‘thistle3’ (**Figure 4**; **Supplementary Dataset S1**) and tested whether they could induce the expression of the nitrate sentinel genes *OsNRT1.1B* and *OsNR1*. We used a rice protoplast transactivation assay to perform *in vivo* validation of the inferred regulatory relationships (**Figure 5**; **Supplementary Figure S4**): a reporter plasmid harboring the mEGFP gene under the control of the promoter of a putative target gene was co-transfected with an expression vector harboring the coding sequence of one of the selected transcription factor downstream of a constitutive promoter (p35s).

We found two transcription factors that strongly induced the expression of *OsNR1* and *OsNRT1.1B*: ETHYLENE INSENSITIVE3 (EIN3)-LIKE1 (OsEIL1)/MAHOHUZI6 (MHZ6) (Os03g20790) and REGULATOR OF LEAF INCLINATION1 (OsRLI1)/HIGHLY INDUCED BY NITRATE GENE1 (HINGE1) (Os04g56990) (**Figure 5**; **Supplementary Figure S4**).

**Figure 6:**
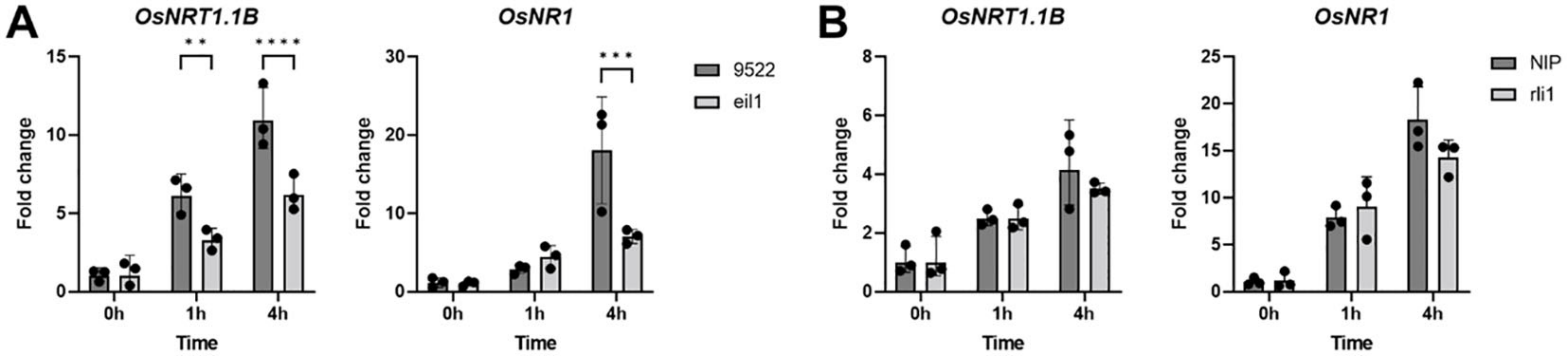
Gene expression of OsNRT1.1B and OsNR1 in osrli1 **(A)** or oseil1 **(B)** mutants or their respective wild-type background in roots of rice supplemented with NO_3_ in the form of KNO_3_. Significance was determined by a one-way ANOVA followed by a Tukey’s post-hoc test (**p < 0.01; ***p < 0.001; ****p < 0.0001).

To further investigate the role of OsRLI1 and OsEIL1 in rice nitrate response, we generated or acquired the mutant rice lines of *oseil1* and *osrli1*. Both mutants showed a small increase in lateral root number and primary root length, but this phenotype was independent of the different nitrogen treatments (**Supplementary Figure S5**). To assess the importance of the transcription factors for the nitrate response, we treated the mutants with nitrate and tracked *OsNRT1.1B* and *OsNR1* expression over time (**Figure 6**). The expression of *OsNRT1.1B* and *OsNR1* was less induced by nitrate in the *oseil1* mutant background than in the wild-type line (**Figure 6A**), which further supports a role of *OsEIL1* for the induction of nitrate responsive genes and hence in the nitrate regulatory pathway. In contrast, we did not detect a significant difference of the nitrate responsive genes in the *osrli1* background (**Figure 6B**).

## Discussion

### Co-expression network identifies novel candidates in nitrogen signaling

In this study, we provided a detailed overview of the transcriptional response of rice in roots and shoots to different nitrogen forms and generated a resource with the expression profile of any rice gene of interest in response to nitrate, ammonium, or the combination of both (all expressions profiles are available on https://www.psb.ugent.be/shiny/rice-response-to-nitrogen/). We used this dataset to generate a co-expression network, and identified clusters with a specific response to nitrogen in both roots and shoots. Furthermore, the co-expression network created the possibility to infer putative transcription factors/target genes relationships. As the different nitrogen treatments lead to distinct variations due to unique interactions, we anticipated uncovering otherwise overlooked regulatory relationships. We illustrated this by identifying new transcription factors with a role in nitrate signaling and showing the potential effect of two transcription factors, OsRLI1 and OsEIL1, on the induction of nitrate response.

OsRLI1 is a transcription factor involved in phosphate starvation signaling (Zhang et al., 2021). As a matter of fact, nitrate is known to affect the phosphate signaling pathway (Hu et al., 2019). Supporting this, our co-expression network revealed that *OsRLI1* expression is correlated with the expression of several phosphate-starvation signature induced genes: *INOSITOL-3-PHOSPHATE SYNTHASE ISOZYME1* (*OsIPS1*) (often used as a phosphate starvation reporter (Hou et al., 2005; Wang et al., 2009a; Dai et al., 2012)), *1-AMINOCYCLOPROPANE-1-CARBOXYLIC ACID SYNTHASE* (*OsACS*) [involved in tolerance to phosphate starvation in rice (Lee et al., 2019)], *SPX-MAJOR FACILITY SUPERFAMILY2* (*OsSPX-MSF2*) (involved in phosphate signaling/transport and induced by phosphate starvation (Wang et al., 2012)), and finally *PHOSPHATE STARVATION RESPONSE2* (*OsPHR2*) which is the main regulator of phosphate starvation responses (Zhou et al., 2008) and inducer of *OsRLI1* (Zhou et al., 2008; Wu and Wang, 2011; Zhang et al., 2021). *OsRLI1* is moreover a close homologue of *OsPHR2* and *AtPHR1* and interacts just as these with SPX-domain containing proteins (Puga et al., 2014; Wang et al., 2014; Ruan et al., 2018; Zhang et al., 2021). OsPHR2 binds to OsSPX1/2/4 upon high phosphate. A low cellular inositol phosphate level, which depends on the phosphate level of the cell, disrupts the SPX retention of OsPHR2 which then is free to migrate to the nucleus where it binds to phosphate starvation inducible genes promoters (Wild et al., 2016; Crombez et al., 2019; Hu et al., 2019). At least the interaction with OsSPX4 depends also on nitrate levels: the transceptor OsNRT1.1B can promote OsSPX4 protein degradation in a nitrate-dependent manner, impacting directly the phosphate signaling pathway (Hu et al., 2019). OsRLI1 was shown to be induced by nitrate to induce the phosphate starvation response and finetune the N-P balance (Zhang et al., 2021). Our results show that it may also induce nitrate responsive genes, further complicating the phosphate-nitrate crosstalk.

OsEIL1 is a transcription factor involved in ethylene signaling (Yang et al., 2015a, Yang et al., 2015b) and regulates various genes such as transcription factors and metabolic genes (Dolgikh et al., 2019) and hormonal pathways (Chang et al., 2013). Here, we showed that *OsEIL1* upregulation by nitrate correlates with *OsNRT1.1B* induction in rice. In Arabidopsis, nitrate induces ethylene production via induction of 1‐aminocyclopropane‐1‐carboxylic acid (ACC) synthases (ACS) and ACC oxidases (ACO), key enzymes in the ethylene biosynthesis pathway (Kende, 1993; Khan et al., 2015). Moreover, nitrate-induced expression of *NRT1.1* requires ethylene signaling (Tian et al., 2009), but it is not known how these pathways exactly connect to each other. Additionally, certain nitrate transporters were shown to be directly controlled by ethylene (Zheng et al., 2013; Zhang et al., 2014). As in Arabidopsis, multiple *ACS* genes are in our dataset induced upon nitrate in our rice dataset, including *OsACS2*, *OsACS5* and *OsACS6*, supporting a comparable pathway in rice and Arabidopsis. However, the absence of binding motifs for the OsEIL1 transcription factor (Hiraga et al., 2009) or ethylene response factors ERFs (Ohme-Takagi and Shinshi, 1995) in the promoters of *OsNRT1.1B* and *OsNR1* argue for an indirect impact on these genes by OsEIL1. Still, our results show that *OsEIL1* is not only able to – possibly indirectly – induce *OsNRT1.1B* and *OsNR1*, but also that *OsEIL1* is important for the nitrate-induced expression of those genes, featuring OsEIL1 as a central transcription factor in the ethylene signaling-dependent nitrate response in rice.

### Ammonium as a signal?

While we focused on the nitrate-specific clusters to investigate new candidate regulators, other parts of the co-expression network can be explored as well. For instance, the ammonium-specific cluster may provide valuable insights into ammonium signaling, although this could be more challenging due to the generally slower transcriptional response compared to nitrate. This slow transcriptional response indicates that ammonium does not directly activate a transcriptionally regulated signaling pathway. Still, ammonium is suggested to be signaling molecule (Liu and von Wirén, 2017). Bacteria have even been shown to possess an ammonium-sensing histidine kinase (Pflüger et al., 2018, Pflüger et al., 2024), but similar mechanisms have not yet been demonstrated in plants. Interestingly, the bacterial sensor is part of the ammonium transporter/methylamine permease/Rhesus family, which also includes plant AMT proteins that have been proposed to function as ammonium receptors (Liu and von Wirén, 2017). The fact that ammonium does not induce rapid transcriptional changes in rice does not exclude that ammonium can act as a potential signaling molecule via another biochemical pathway and indirectly trigger a transcriptional response. In this respect, it is important to note that we observed a strong transcriptional response starting 1 hour after treatment, with a considerable number of transcription factors identified in the ammonium-specific clusters, including for example *MONOCULM1* (*OsMOC1*, *Os04g35250), OsNAC5* (*Os11g08210*) and *OsNLP6 (Os02g04340)* that showed high expression levels (FC > 8) after 1 hour of treatment. Interestingly, *OsNLP6* is a homolog of *OsNLP1*, *OsNLP3*, and *OsNLP4* which are all known for their implication in nitrate and ammonium responses or in nitrogen use efficiency (Hu et al., 2019; Alfatih et al., 2020; Wang et al., 2021; Wu et al., 2021). *OsNLP6* is only known for having a very low basal expression and not responding to various stress tested in previous studies, but was never characterized further (Jagadhesan et al., 2020; Wu et al., 2021). The high expression of *OsMOC1* is somewhat surprising as it is mainly known for its critical role in regulating tiller number and plant architecture (Liao et al., 2019). Finally, *OsNAC5* is an abiotic stress-responsive gene (Takasaki et al., 2010), which might indicate that a stress induce the transcriptional response. Ammonium is known to affect rapidly the internal and external pH of roots, which may be the chemical cue resulting in this response (Jia et al., 2020; Motte and Beeckman, 2020). We also observed that ammonium upregulated alanine aminotransferases expression, indicating an accumulation of alanine in planta. Such responses are usually observed in stress conditions to store nitrogen and to provide energy and reductants under for instance anoxia situations in the cell (Vanlerberghe et al., 1991; Miyashita et al., 2007). Alanine biosynthesis is a known ammonium detoxification process with alanine serving as a nitrogen store (Esteban et al., 2016). In Arabidopsis roots, hypoxia induces *AlaAT1* and *AlaAT2* as early as 2h after stress application with a peak at 8h, followed by a decrease after 24h, which corresponds to what we and others observed in rice upon ammonium treatment (Miyashita et al., 2007) and was also observed in maize (Muench et al., 1998). Gene ontology enrichment for the ammonium-specific cluster (‘darkslateblue’) revealed an increase in proton related ATPase activity terms potentially indicating a response to counteract cytoplasmic acidification caused by ammonium uptake, thereby contributing to ammonium tolerance in rice. The enrichment of the pyruvate metabolic process term suggests a higher demand for energy production or amino acid biosynthesis, as pyruvate is a central metabolite connecting glycolysis, the TCA cycle, and the amino acid synthesis pathways. Overall, this suggests that the response is more likely related to acidification or stress rather than ammonium acting as a signaling molecule. In any case, the poor overlap in response to ammonium in the shoot and root supports a local effect.

### Synergistic effects: dual action or mitigation of stress?

Both in our and previous studies, co-application of ammonium-nitrate resulted in more growth compared to both forms individually (**Figure 1**) (Kronzucker et al., 1999). Our data showed a broader transcriptional response to the combined nitrogen treatment, encompassing responses that are otherwise only elicited by either ammonium or nitrate alone. This is particularly clear in the cluster analysis, where the ammonium-nitrate profile closely follows either the ammonium or nitrate expression patterns, but rarely exhibits a distinct profile. Hence, the combined provision may elicit a dual action that translate into improved growth. This was specifically observed in lateral root density, where the spatial distribution resulting from the combined treatment resembled the cumulative distribution patterns observed under each individual nitrogen form. Additionally, the ammonium treatment resulted in higher leaf chlorophyll content, which is in line with the positive effect of ammonium on photosynthesis activity as reported in Arabidopsis (Sanchez-Zabala et al., 2015). This effect was also observed with the ammonium-nitrate combination, but not with nitrate alone, further illustrating that the action of one of the forms is preserved within the combined treatment.

An alternative explanation for the differences in growth between co-application and single application is that the provision of only one nitrogen form could trigger a stress response, which is absent when both forms are present. Indeed, despite rice being considered as an ammonium-tolerant plant, we observed that ammonium supplementation alone reduces the size of the rice root system, a phenotype typically associated with ammonium toxicity (Liu and von Wirén, 2017). Accumulation of chlorophyll is in Arabidopsis associated with a mild ammonium stress (Sanchez-Zabala et al., 2015). Likewise, the ‘yellow3’ co-expression cluster that group genes induced by ammonium but not by ammonium-nitrate shows an oxidative stress signature, while a number of stress-related genes are induced upon ammonium treatment (see above). Hence, while considered to be ammonium tolerant, rice clearly displays toxicity-related phenotypes, as also observed in other recent studies (Jia et al., 2020; Xie et al., 2023; Yan et al., 2023). The presumed ammonium tolerance likely originates from observations of paddy field-grown rice, where ammonium is partially converted to nitrate, and rice at the end perceives both ammonium and nitrate. Furthermore, genes in the ‘indianred3’ and ‘plum4’ clusters that are exclusively induced by nitrate only and by none of the other treatments are primarily linked to iron homeostasis and transport as illustrated by the GO enrichment. Such genes, including *OsIRO2, OsIRO3*, *OsNRAMP1*, *OsPOT*, *OsOPT7* and *OsMIR* are typically upregulated upon iron starvation (Zheng et al., 2009; Zhang et al., 2017), which is known to occur when nitrate is the sole nitrogen form provided (Chen et al., 2018). Hence, the observed improvement in growth with the combined treatment may be attributed to the mitigation of stress effects that are typically induced by the individual nitrogen forms.

### Nitrogen network for data mining

By focusing on a few nitrate-specific clusters, we demonstrated that our dataset, which includes responses to both ammonium and nitrate, can be utilized to identify candidate transcription factors involved in nitrogen signaling. Other clusters with different nitrogen response profiles presented in this study can be investigated as well, either to identify novel regulators or to predict functions for unknown genes. For instance, uncharacterized putative transporter encoding genes that were identified as strongly co-expressed with nitrate transporters in our network might encode transporters with a role in nitrate transport. Overall, our present study provides the research community with an extensive dataset describing how rice, a major staple crop, responds at the transcriptional level to two main nitrogen feedstocks. A better understanding of how plants sense, take up and process the two main forms of nitrogen provided by fertilization is an important field of study within the contemporary context of the increasing need to breed crop plants with enhanced nitrogen use efficiency.

## Material and methods

### Root and shoot treatment and sampling for transcriptomics

Rice seedlings [Oryza sativa Nipponbare cultivar (#GSOR100, USDA-ARS)] were dehulled and sterilized with ethanol 70% for 5 minutes, followed by immersion in bleach 6% with Tween-20 for 30 minutes. Seedlings were imbibed by immersion in sterile water for 12h to synchronize germination at 30 degrees. Germinating seeds were transferred on a hydroponic system, and roots were immersed in a nitrogen-free basal salt medium composed of K_2_SO_4_ 0.7mM, KH_2_PO_4_ 0.3mM, CaCl_2_.2H_2_O 1mM, MgSO_4_.7H_2_O 1mM, Na_2_SiO_3_.9H_2_O, Na_2_-Fe-EDTA 20µM for macronutrients, and MnCl_2_.4H_2_O 9µM, Na_2_MoO_4_.2H_2_O 0.39µM, H_3_BO_3_ 20µM, ZnSO_4_.7H_2_O 0.77µM, CuSO_4_.5H_2_O 0.32µM for micronutrients (pH 5.8). Seedlings were then transferred to a growth cabinet in the dark at 30 degrees for 3 days in a randomized block design. The light was then turned on after 72h and let on for 48h before treatment occurred. Nitrogen treatments consisted of injection with 5mM KNO_3_ (5mM NO_3_^-^ treatment), 2.5mM (NH_4_)_2_SO_4_ + 2.5mM K_2_SO_4_ (5mM NH_4_^+^ treatment), 2.5mM KNO_3_ + 1.25mM (NH_4_)_2_SO_4_ + 1.25mM K_2_SO_4_ (2.5mM NH_4_^+^ and 2.5mM NO_3_^-^ treatment) or 2.5mM K_2_SO_4_ (mock treatment) in this basal medium. K_2_SO_4_ was used to balance potassium (K^+^) equimolarly to 5mM in each of the treatments. Rice seedlings were extracted 15min, 1h, 2h, 4h, 12h, 24h and 48h after nitrogen treatments. A supplemental control without treatment was extracted at the 0h time point in 3 biological replicates for roots and shoots, to estimate the impact of the manipulation of the samples (referred to as “Control 0h”). At the extraction time-point, shoots and roots were cut with a razor blade and frozen in liquid nitrogen. The remaining seeds were discarded. Three different boxes were used for each treatment and for each time-point, for a total of 87 boxes. At least 10 germinated seedlings were sampled per box.

### Root and shoot phenotyping

For the phenotyping experiments, the same procedure as described above was followed but seedlings were let grown in the hydroponic media for 10 days after treatment and the medium was refreshed daily. Chlorophyll was extracted with DMSO and measured by absorbance at 663nm (Chlorophyll A) and 645nm (Chlorophyll B). Chlorophyll content was measured as:


Chlorophyll A(mmol=l=g) = (½Abs at 663nm=½75:05*1)=g of fresh leaves

Chlorophyll B(mmol=l=g) = (½Abs at 645nm=½47:0*1)=g of fresh leaves

### RNA extraction

Frozen roots and shoot samples were grinded with one 3mm metal bead into Eppendorf tubes. RNA was extracted with Trizol (Life Technologies) and the RNeasy Mini Kit (Qiagen) following the manufacturer instructions. An extra DNase step was performed with RNase-Free DNase Set (Qiagen). RNA samples were resuspended in RNAse free water. RNA concentration and purity were determined spectrophotometrically using the Nanodrop ND-1000 (Nanodrop Technologies) and RNA integrity was assessed using a Bioanalyzer 2100 (Agilent).

### RNA-seq library preparation

The sequencing and library preparation was performed by the VIB Nucleomics Core Facility (Leuven, Belgium; www.nucleomics.be). Per sample, 500ng of total RNA was used as input. Using the Illumina TruSeq^®^ Stranded mRNA Sample Prep Kit (protocol version: Part # 15031047 Rev. E - October 2013), poly-A containing mRNA molecules were purified from the total RNA input using poly-T oligo-attached magnetic beads. In a reverse transcription reaction using random primers, RNA was converted into first strand cDNA and subsequently converted into double-stranded cDNA in a second strand cDNA synthesis reaction using DNA PolymeraseI and RNAse H. The cDNA fragments were extended with a single ‘A’ base to the 3’ ends of the blunt-ended cDNA fragments after which multiple indexing adapters were ligated introducing different barcodes for each sample. Finally, PCR enrichment was conducted to enrich those DNA fragments that have adapter molecules on both ends and to amplify the amount of DNA in the library. Sequence-libraries of each sample were equimolarly pooled and sequenced on Illumina NextSeq 500 (High Output, 75 bp, Single Reads, v2). The raw transcriptomic data (*fastq* files) have been deposited in the functional genomics data collection ArrayExpress under the accession number E-MTAB-13146.

### Sequence mapping

All analyses were done on the VIB-UGent Plant System Biology Galaxy platform (Afgan et al., 2018). The Trimmomatic tool (Bolger et al., 2014) was used to trim the reads for low-quality read-ends with the following options: raw fastq file, type TrueSeq3 adapter sequences. Data quality was assessed with the FastQC tool before and after trimming with the Trimmomatic tool. The output of Trimmomatic was processed by the Salmon tool (Patro et al., 2017). Salmon was used for transcript-level quantification estimates of RNAseq data. The reads were mapped on the coding sequences of release 7 of the MSU Rice Genome Annotation Project (Kawahara et al., 2013) with the following options: stranded reads and reads derived from the reverse strand, with an Incompatible Prior setting of 1x10^-20^. Salmon acts in two steps: the indexation of the reference genome (Oryza sativa japonica v7JGI) and the mapping of the reads trimmed by Trimmomatic to this reference genome, followed by their quantification. The output is an estimated number of reads in transcript per millions. The package txtimport 1.14.0 (Soneson et al., 2015) in the R Statistical software version 3.4.3 was used to process the Salmon output data (transcript-level abundance) and summarize it into matrices of counts of reads/fragments (gene-level abundance).

### Differential expression analysis

#### DESeq2 data preparation and cleaning

The txtimport output was then processed with the DESeq2 version 1.26.0 package for differential analysis (Love et al., 2014). A DESeqDataSet was created using the function ‘DESeqDataSetFromTximport’ with a design (~time + treatment + time:treatment), with time and treatment as categorical variables. We then used the DESeq() function to estimate size factors and dispersion values, fit a negative binomial model to the count data, and perform differential gene expression analysis. The resulting DESeqDataset was normalized using the varianceStabilizingTransfomation() (VSD) function. A heatmap of sample-to-sample distance comparison was built for roots and shoots independently to identify outliers samples, using the VSD-transformed data as recommended by the WGCNA developers. Two outliers were detected with the heatmap: one outlier in the roots (2h after NH_4_^+^ treatment, replicate 3) and one in the shoots (1h after NO_3_^-^ treatment, replicate 2). These samples were discarded for further analysis. The samples correlation was assessed by PCA analysis once outliers were removed (**Supplementary Figures S6**, **S7**) and illustrate a good clustering of the samples.

#### Pair-wise differential analysis

For the pair-wise differential analysis, the same DESeqDataSet was used as input; the DESeq() function was used repeatedly with contrasts set manually between each treatment and the control for each time points independently. Genes with an absolute fold-change > 2 and an FDR < 0.05 were considered as differentially expressed.

### Gene co-expression construction

The gene co-expression network and clusters were built using the WGCNA package (Langfelder and Horvath, 2008). We used the varianceStabilizingTransformation() (VSD) function of the package DESeq2 to transform and normalize the DESeqDataSet data described above without the outliers, as recommended for big experiments containing more than 100 samples, and averaged the 3 biological samples per treatment, per time-point. Only genes with more than 5 counts in at least 2 repetitions per treatment per time point were kept, removing non or very lowly expressed genes. This first threshold reduced the total number of genes to around 26000 for roots and shoots. For computational reasons and to remove noise background, a second threshold removing the 30% least-varying genes based on their expression variance between the treatments as recommended by the WGCNA developers was applied. The final input for the gene co-expression network construction was 18343 genes for the shoots and 18457 genes for the roots. DatasetGene connectivity was determined with a power β (soft thresholding) of 7 for the roots and to 8 for the shoots, chosen with the function pickSoftThreshold() with the following options: networkType = “signed hybrid”, corFnc = “bicor”, maxPOutliers = 0.02. The function ‘adjacency()’ was used with the same options. The options used to design the network with the function cutreeDynamic were deepSplit = 3, and minModuleSize = 20. For every cluster generated, a cluster eigengene is computed; this eigengene (first principal component of a cluster) can be seen as representative of all the genes that compose the cluster. Eigengenes with a correlation with another eigengene higher than 80% (R2 = 0.8) were merged into one cluster. Network visualization was done with Cytoscape 3.7.2 (Shannon et al., 2003)

### Gene ontology enrichment analysis

To identify enriched biological processes, molecular functions, and cellular components within co-expression clusters, a Gene Ontology (GO) enrichment analysis was performed using the GO enrichment tool of the Plaza Monocots 4.0 Platform (Van Bel et al., 2018) using the Locus ID and the publicly available Rice v7.0JGI database with the whole annotated genome as the reference set. The significance threshold for enriched GO terms was set at a p-value of 0.01.

### Plasmid construction

Transcription factor coding sequences were isolated by PCR from rice shoots or root cDNA and used to generate the ‘inducers plasmids’. Promoter sequences of the target genes were isolated from genomic DNA and correspond to the -2000bp sequence upstream of the start codon of the target gene or were limited by the presence of another gene downstream and used to generate the ‘target plasmids’. The plasmids were constructed with the Golden Gateway assembly system: in the inducer plasmids, the coding sequences of the transcription factors were combined with a constitutive promoter (p35s) followed by a nuclear localization sequence. A NOST terminator was placed downstream of the gene coding sequence. In the target plasmids, the genes promoters were cloned upstream of a nuclear localization sequence followed by the fluorescent protein mEGFP coding sequence and a NOST terminator. The inducers plasmids structure can be summarized as “p35s::NLS::transcription-factor-CDS::NOST”. The target plasmids structure can be summarized as “gene-promoter::NLS::mEGFP::NOST”. Sequences were validated by sequencing (Eurofins Genomics, Belgium) and reference sequences were extracted from the Plaza Monocots 4.0 Platform (Van Bel et al., 2018). The list of primers used for the genes coding sequences and promoter isolation is available in **Supplementary Table S1**.

### Extraction and transformation of rice protoplasts

14-days old rice seedlings (#GSOR100 USDA-ARS) grown in the dark in sterile vitro-vent boxes on a solid media containing 0.305g/l Murashige & Skoog Modified Basal Salt Mixture Nitrogen-free salts (Phytotech Labs #M407), 0.6mM KH_2_PO_4_, 9.4mM K_2_SO_4_, 1mM NH_4_NO_3_, 1.6mM Na_2_SiO_3_.9H_2_O, 8g/l agar and 0.025g/l MES at pH 5.7, were harvested by cutting the stem above the seed and the aerial part kept for protoplast isolation. The protoplasts extraction and transformation followed the protocol described in other studies with few adaptations (Abel and Theologis, 1994; Yoo et al., 2007; Zhang et al., 2011). Briefly, once extracted, the protoplasts were mixed with different combinations of one inducer plasmid and one target plasmid. Addition of PEG-4000 to the mix induced the transient transformation of the protoplasts which assimilated the different combinations of the two types of plasmids, and transformation was stopped after 15 minutes. After incubation overnight, the protoplasts in solution were distributed in a 90-well plate and mEGFP fluorescence intensity (excitation: 488nm, emission: 522nm) was measured by confocal microscopy.

### Generation of the oseil1 and osrli1 mutants

The OsEIL1 knock-out mutant was generated in a *Japonica* variety Wuyunjing-7 (9522) using the CRISPR-Cas9 technique, while OsRLI1 knock-out mutant is *Japonica* variety Nipponbare background and was generated in a previous study (Ruan et al., 2018). Homozygous mutant lines were used for subsequent analysis.

### Phenotyping and RT-qPCR of the oseil1 and osrli1 mutants

Rice seeds of wild-type and mutant lines were sterilized with 70% (v/v) ethanol for 1 min, followed by 30% (v/v) sodium hypochlorite solution for 30 min. Seedlings were imbibed by immersion in sterile water for 12h to synchronize their germination and let grown in the dark on nitrogen free solution for 3 days, and then transferred to the growth chamber (30 degrees, continuous light) for another 3 days. Seedlings with ~2 cm seminal root were selected for different nitrogen treatments with modified Kimura B solution: high nitrogen (1.5 mM (NH_4_^+^)_2_SO_4_, or 3 mM KNO_3_^-^, HN) and nitrogen free (- N or N-free). The time course started at the moment of the transfer. 20 seedlings roots per technical replicate where harvested, and samples were processed as described above for the transcriptome experiment. The RNA was synthetized into cDNA, and the primers presented in **Supplementary Table S1** were used for the RT-qPCR as previously described (Xie et al., 2023)

### Phenotyping of the oseil1 and osrli1 mutants

Geminated rice seedlings were first grown in water for 3 days in a growth chamber under a photoperiod of 14 h light (200μmol m-^2^ s-^2^ light density and 70% humidity) and a temperature of 28 degrees, and rice seedlings with ~2 cm long seminal root were then transferred to the hydroponic culture supplied with modified Kimura B solution (500 mL volume for each cup with 10 seedlings) for different nitrogen treatments. For nitrogen -free treatment, nitrogen sources (NH_4_)_2_SO_4_ and KNO_3_ was replaced with K_2_SO_4_ at a concentration of 1.5 mM; for NH_4_^+^ treatment alone, KNO_3_ was replaced with K_2_SO_4_ at the same concentration; for NO_3_^-^ treatment alone, (NH_4_)_2_SO_4_ was replaced with 3 mM KNO_3_. The 2-[morpholino]ethane sulfonic acid (MES) was supplied to hydroponic cultures to buffer pH of the medium when mentioned. The rice seedlings were treated for 4 days, and the nutrient solution was renewed every two days.

## Data Availability

The datasets presented in this study can be found in online repositories. The names of the repository/repositories and accession number(s) can be found in the article/**Supplementary Material.**

## Supplementary material

Supplementary Figure 1 Number of differentially expressed genes (FDR < 0.05, absolute fold-change > 2), for each time point in the roots. The bar represents the number of genes present at the intersection indicated by the dot in the bottom of the graph. The Gene/Treatments graph represent the total number of genes differentially regulated per treatment. Brown: genes differentially expressed by NH_4_NO_3_ only. Yellow: genes differentially expressed by NH_4_^+^ only. Red: genes differentially expressed by NO_3_^-^ only. Grey: other combinations as presented below the graph.

Supplementary Figure 2 Number of differentially expressed genes (FDR < 0.05, absolute fold-change > 2), for each time point in the shoots. The bar represents the number of genes present at the intersection indicated by the dot in the bottom of the graph. The Gene/Treatments graph represent the total number of genes differentially regulated per treatment. Brown: genes differentially expressed by NH_4_NO_3_ only. Yellow: genes differentially expressed by NH_4_^+^ only. Red: genes differentially expressed by NO_3_^-^ only. Grey: other combinations as presented below the graph.

Supplementary Figure 3 Number of differentially expressed genes (FDR < 0.05, absolute fold-change > 2), for each time point in the roots **(A)** and shoots **(B)**. The histogram plot represents the number of genes present at the intersection indicated by the dot in the bottom of the graph. The Gene/Time points graph represent the total number of genes differentially regulated per treatment. Blue: genes that are differentially regulated from the first time point (15 minutes after treatment) after treatment and that remain differentially regulated at each time point until the end of the time course (48h after treatment). Yellow: genes that are differentially regulated from 1h after treatment and that remain differentially regulated at each time point until the end of the time course (48h after treatment).

Supplementary Figure 4 WGNCA co-expression clusters in the root. Overview of the expression profile of all clusters. The average expression of all the genes composing the cluster is presented in red, individual gene expression is shown in black. Within each plot, the profile of mock, ammonium (NH4), ammonium-nitrate (NN) and nitrate (NO3) is shown from left to right. The name and number of genes per cluster is indicated at the top of each plot.

Supplementary Figure 5 WGNCA co-expression clusters in the shoot. Overview of the expression profile of all clusters. The average expression of all the genes composing the cluster is presented in red, individual gene expression is shown in black. Within each plot, the profile of mock, ammonium (NH4), ammonium-nitrate (NN) and nitrate (NO3) is shown from left to right. The name and number of genes per cluster is indicated at the top of each plot.

Supplementary Figure 6 Screenshot of Shiny app enabling access to the rice gene expression profiles in response to different nitrogen treatments and the co-expression analysis. 1: User selected gene of interest. 2: Option to select a threshold for the co-expression coefficient in the table 5 and 6. 3: Gene expression profile in response to different forms of nitrogen over a time-course in the roots or the shoots. 4: Eigengene of the WGCNA cluster of the selected gene in the roots or the shoots. 5,6: List of genes co-expressed with the gene of interest in the roots or the shoots. The co-expression coefficient corresponds to the adjacency table (biweight midcorrelation) constructed with WGCNA. Available at https://www.psb.ugent.be/shiny/rice-response-to-nitrogen/.

Supplementary Figure 7 Complete protoplast transactivation assay. Induction of nitrate response genes by the different transcription factors in a rice protoplast transactivation assay. The boxplots show the average mEGFP fluorescence intensity per transfected protoplast (min. 118 protoplasts per condition, average 408) in one well (n=16). Samples (green) are co-transfected with the indicated combinations of inducer and target plasmids. The negative controls are only transfected with the inducer plasmid (blue) or with the target plasmids (red). Significance was determined by a one-way ANOVA followed by a Tukey’s post-hoc test (*** p < 1.10-6, blue: sample versus the transcription factor control, red: versus the promoter of the reporter control).

Supplementary Figure 8 Phenotypes under different nitrogen treatments of oseil1 mutants and osrli1 mutants. (A): Images of the oseil1 mutant and its 9522 background, with measurements of the seminal and lateral roots number. 9522 is the genetic background in which the oseil1 mutant has been constructed. **(B)**: Images of the osrli1 mutant and its NIP background, with measurements of the seminal and lateral roots number. NIP is the genetic background in which the osrli1 mutant has been constructed. The orange dotted line indicates the position of the root tip when the seedlings were transferred to medium supplied with different N. The white dotted line indicates the position of the root tip when the seedlings were treated for 4 days. Different letters correspond to the post-hoc Tuckey’s test significance (p.value=0.05), performed after a two-way ANOVA test, and show significant differences between the samples.

Supplementary Figure 9 Principal component analysis of the roots RNA-seq samples. Principal component analysis of the of the DESeq2 output normalized with the varianceStabilizingtransFormation() function in roots.

Supplementary Figure 10 Principal component analysis of the shoots RNA-seq samples. Principal component analysis of the of the DESeq2 output normalized with the varianceStabilizingTransformation() function in shoots.

Supplementary Table 1 Primers used in this study

Supplementary Data Sheet 1 Genome-wide differential gene expression analysis upon different nitrogen treatments in rice roots

Supplementary Data Sheet 2 Genome-wide differential gene expression analysis upon different nitrogen treatments in rice shoots.

Supplementary Data Sheet 3 Co-expression coefficients between gene pairs corresponding to the adjacency table (biweight midcorrelation) of the roots co-expression network constructed with the WGCNA tool.

Supplementary Data Sheet 4 Co-expression coefficients between gene pairs corresponding to the adjacency table (biweight midcorrelation) of the shoots co-expression network constructed with the WGCNA tool.

Supplementary Data Sheet 5 Gene ontology enrichment of the WGNCA root co-expression clusters.

Supplementary Data Sheet 6 Gene ontology enrichment of the WGNCA shoot co-expression clusters.
