# Supplementary figures and images for: Nitrate and ammonium, the yin and yang of nitrogen uptake: a time-course transcriptomic study in rice
Source: Front Plant Sci. 2024 Aug 23;15:1343073. doi: 10.3389/fpls.2024.1343073 (PMC11377263; doi:10.3389/fpls.2024.1343073)

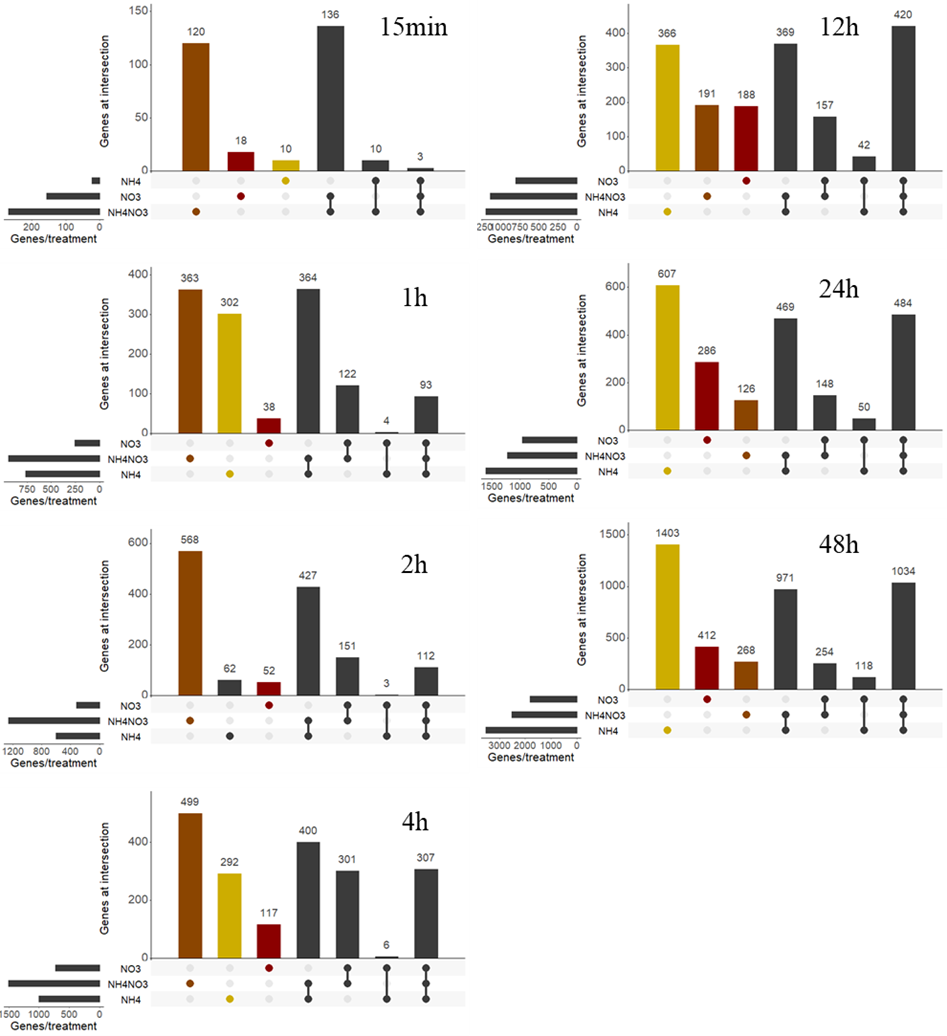

Supplement: Supplementary Figure 1 — Number of differentially expressed genes (FDR < 0.05, absolute fold-change > 2), for each time point in the roots. The bar represents the number of genes present at the intersection indicated by the dot in the bottom of the graph. The Gene/Treatments graph represent the total number of genes differentially regulated per treatment. Brown: genes differentially expressed by NH4NO3 only. Yellow: genes differentially expressed by NH4 + only. Red: genes differentially expressed by NO3 - only. Grey: other combinations as presented below the graph. [file Image1.tif]

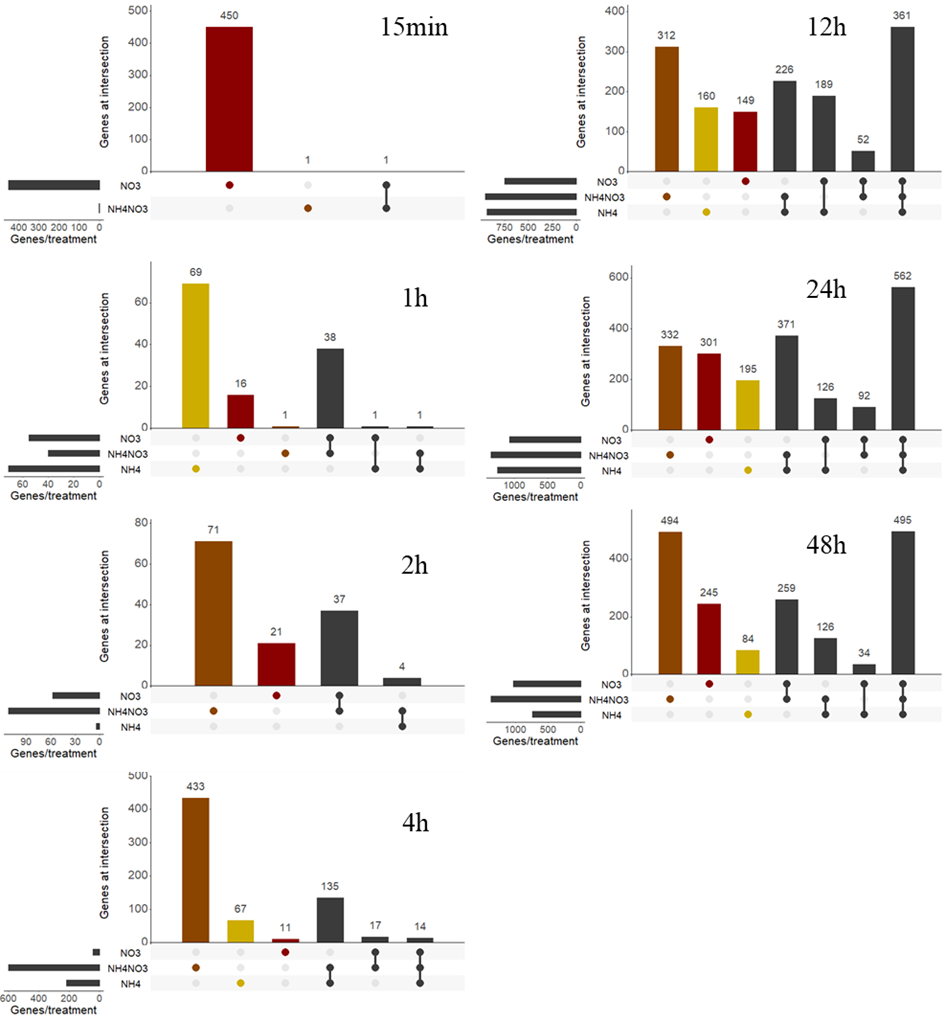

Supplement: Supplementary Figure 2 — Number of differentially expressed genes (FDR < 0.05, absolute fold-change > 2), for each time point in the shoots. The bar represents the number of genes present at the intersection indicated by the dot in the bottom of the graph. The Gene/Treatments graph represent the total number of genes differentially regulated per treatment. Brown: genes differentially expressed by NH4NO3 only. Yellow: genes differentially expressed by NH4 + only. Red: genes differentially expressed by NO3 - only. Grey: other combinations as presented below the graph. [file Image2.tif]

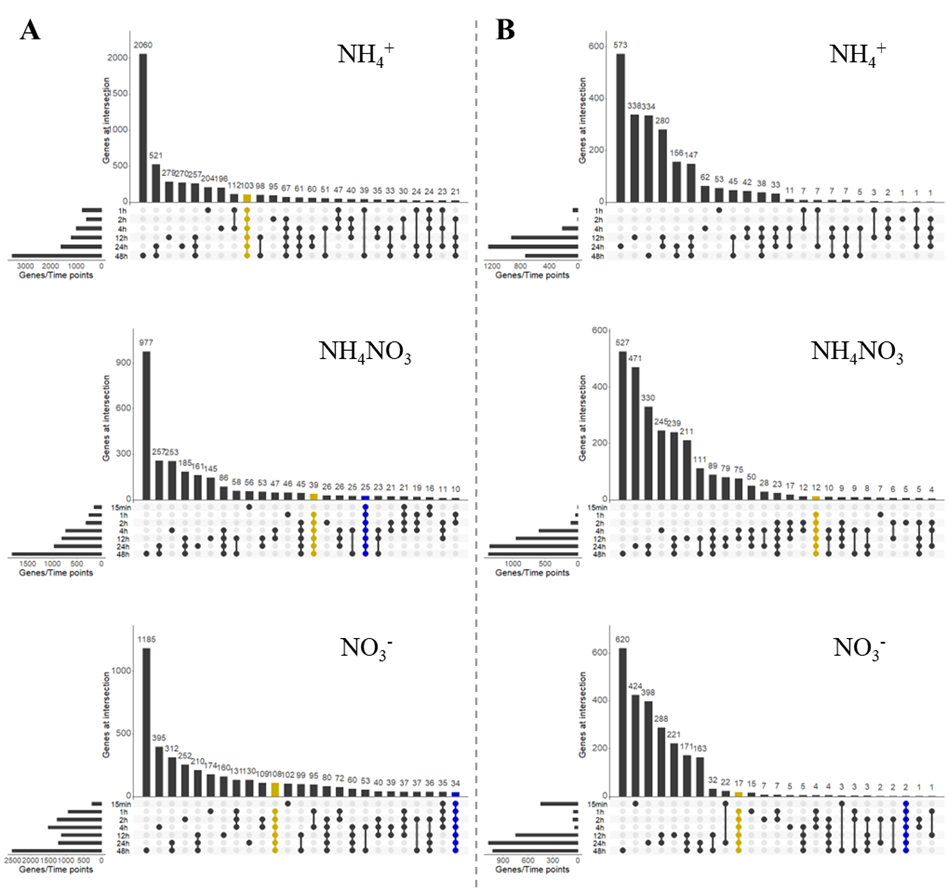

Supplement: Supplementary Figure 3 — Number of differentially expressed genes (FDR < 0.05, absolute fold-change > 2), for each time point in the roots (A) and shoots (B). The histogram plot represents the number of genes present at the intersection indicated by the dot in the bottom of the graph. The Gene/Time points graph represent the total number of genes differentially regulated per treatment. Blue: genes that are differentially regulated from the first time point (15 minutes after treatment) after treatment and that remain differentially regulated at each time point until the end of the time course (48h after treatment). Yellow: genes that are differentially regulated from 1h after treatment and that remain differentially regulated at each time point until the end of the time course (48h after treatment). [file Image3.tif]

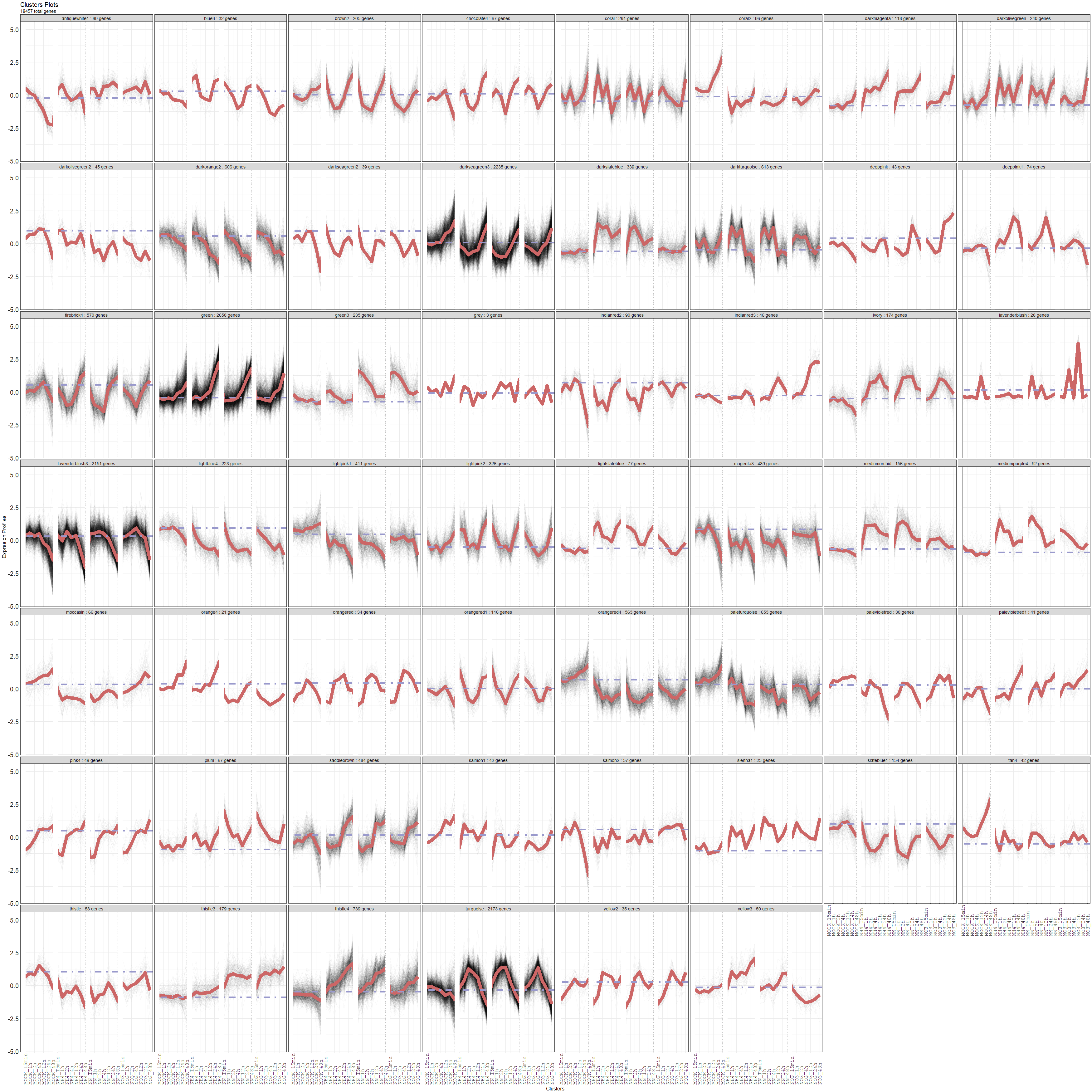

Supplement: Supplementary Figure 4 — WGNCA co-expression clusters in the root. Overview of the expression profile of all clusters. The average expression of all the genes composing the cluster is presented in red, individual gene expression is shown in black. Within each plot, the profile of mock, ammonium (NH4), ammonium-nitrate (NN) and nitrate (NO3) is shown from left to right. The name and number of genes per cluster is indicated at the top of each plot. [file Image4.png]

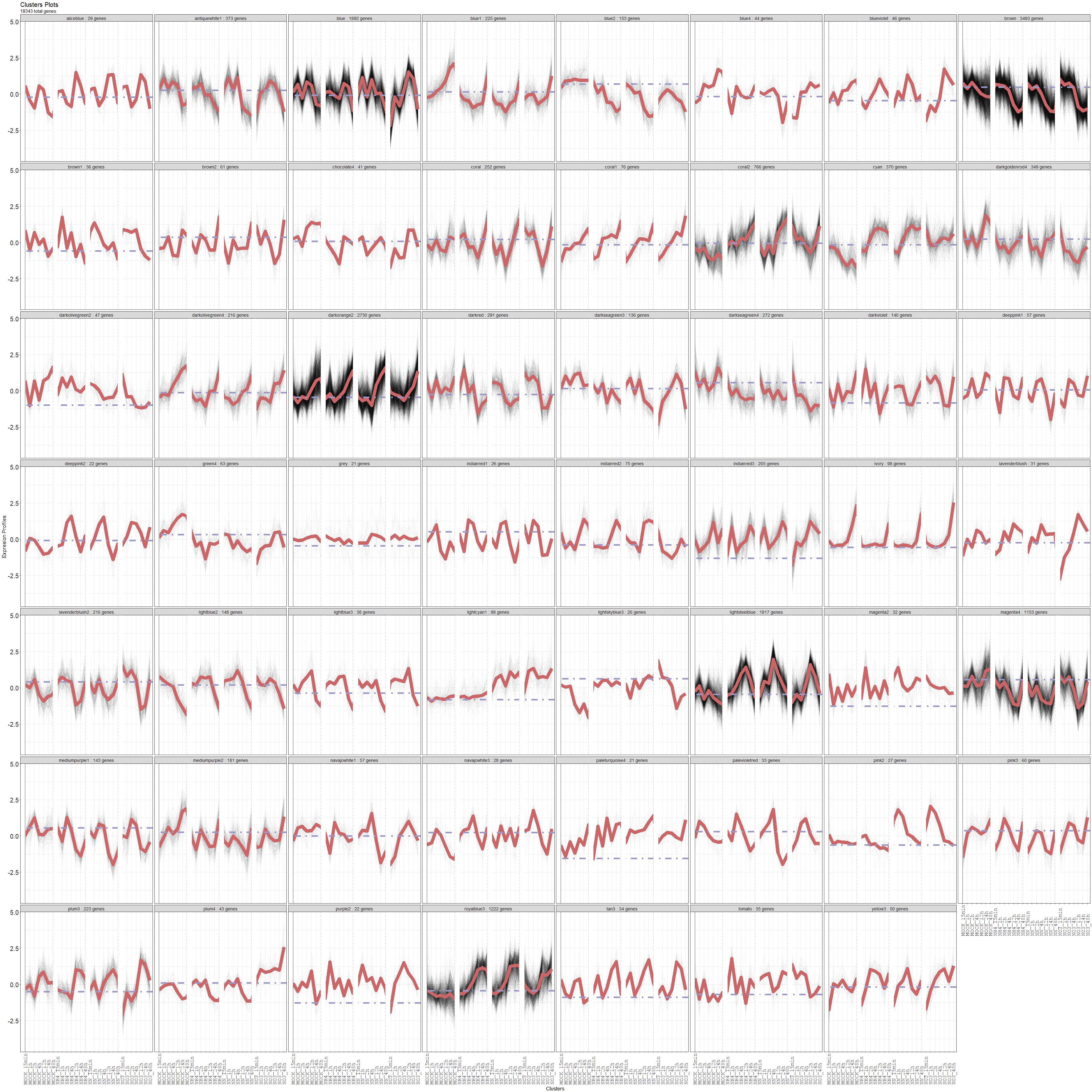

Supplement: Supplementary Figure 5 — WGNCA co-expression clusters in the shoot. Overview of the expression profile of all clusters. The average expression of all the genes composing the cluster is presented in red, individual gene expression is shown in black. Within each plot, the profile of mock, ammonium (NH4), ammonium-nitrate (NN) and nitrate (NO3) is shown from left to right. The name and number of genes per cluster is indicated at the top of each plot. [file Image5.png]

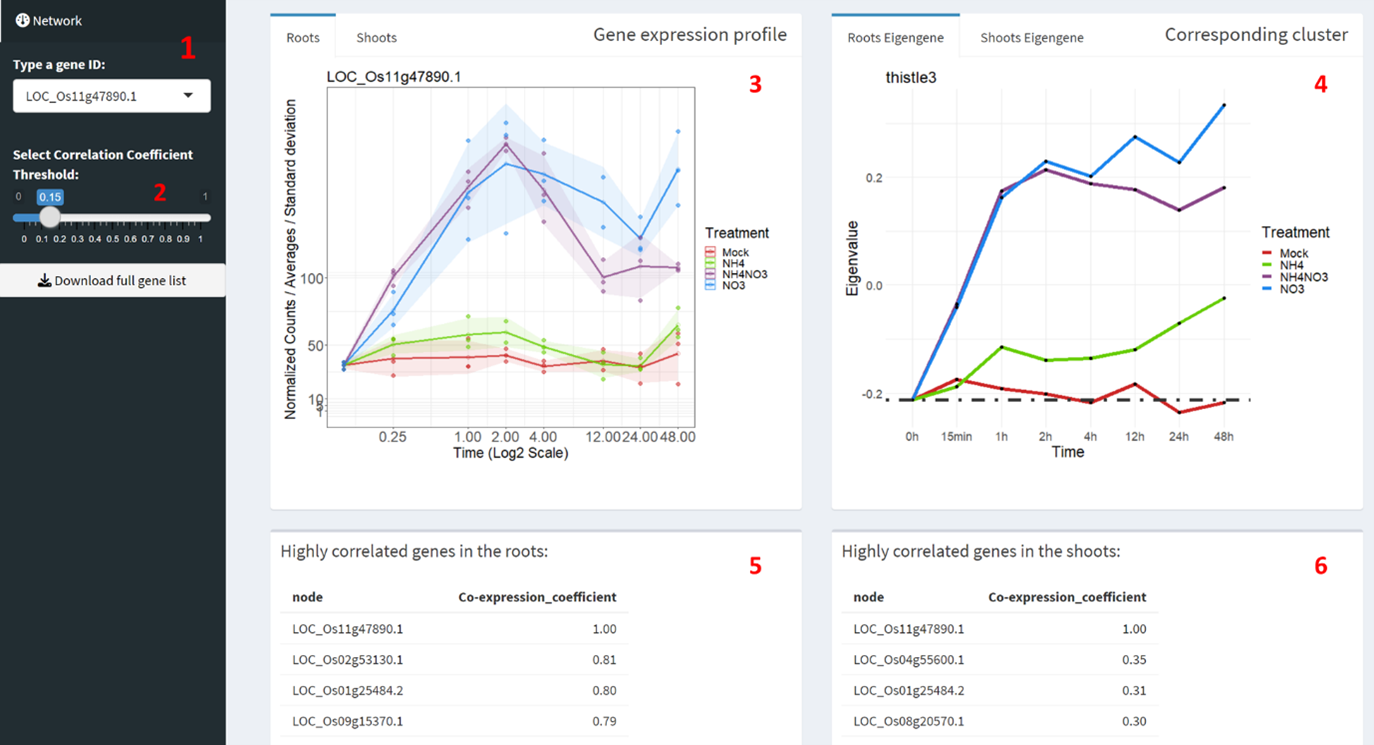

Supplement: Supplementary Figure 6 — Screenshot of Shiny app enabling access to the rice gene expression profiles in response to different nitrogen treatments and the co-expression analysis. 1: User selected gene of interest. 2: Option to select a threshold for the co-expression coefficient in the table 5 and 6. 3: Gene expression profile in response to different forms of nitrogen over a time-course in the roots or the shoots. 4: Eigengene of the WGCNA cluster of the selected gene in the roots or the shoots. 5,6: List of genes co-expressed with the gene of interest in the roots or the shoots. The co-expression coefficient corresponds to the adjacency table (biweight midcorrelation) constructed with WGCNA. Available at https://www.psb.ugent.be/shiny/rice-response-to-nitrogen/. [file Image6.tif]

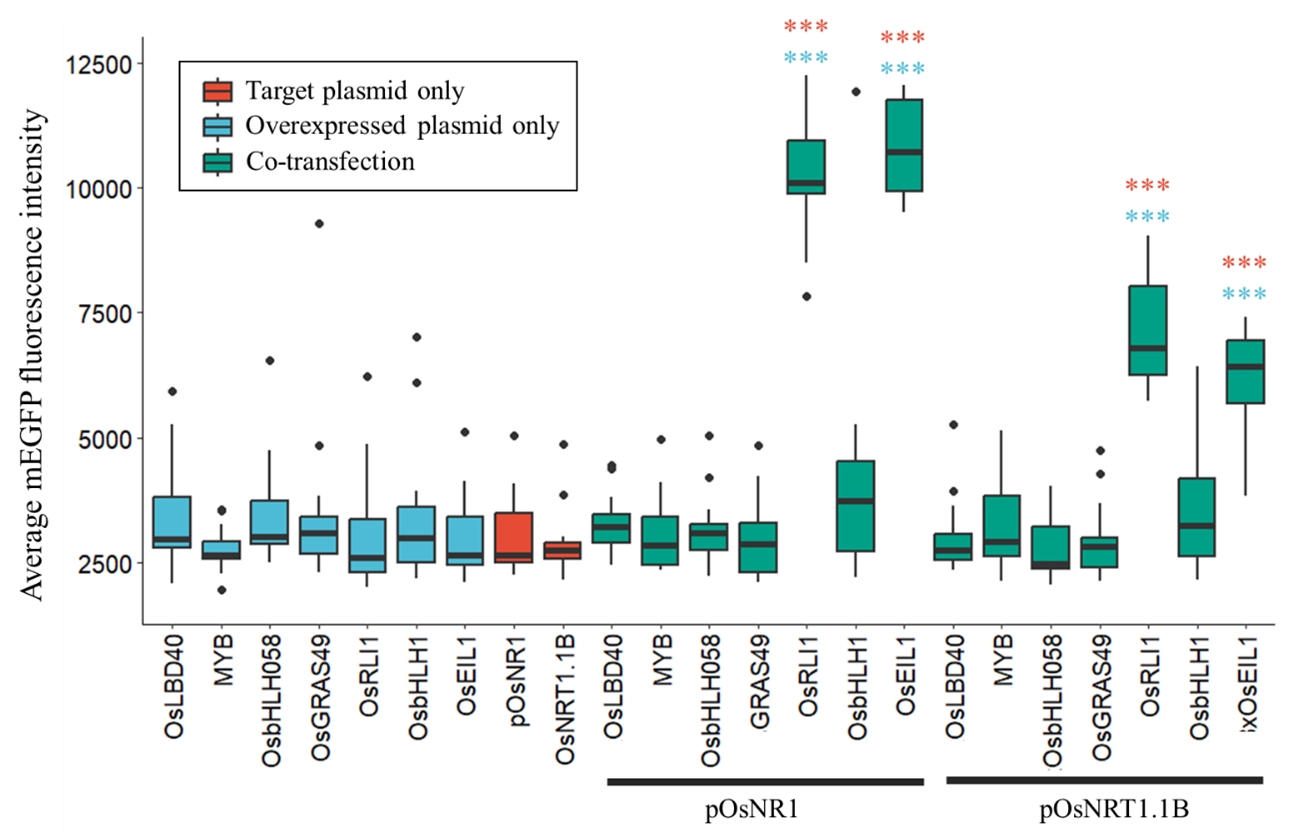

Supplement: Supplementary Figure 7 — Complete protoplast transactivation assay. Induction of nitrate response genes by the different transcription factors in a rice protoplast transactivation assay. The boxplots show the average mEGFP fluorescence intensity per transfected protoplast (min. 118 protoplasts per condition, average 408) in one well (n=16). Samples (green) are co-transfected with the indicated combinations of inducer and target plasmids. The negative controls are only transfected with the inducer plasmid (blue) or with the target plasmids (red). Significance was determined by a one-way ANOVA followed by a Tukey’s post-hoc test (*** p < 1.10-6, blue: sample versus the transcription factor control, red: versus the promoter of the reporter control). [file Image7.tif]

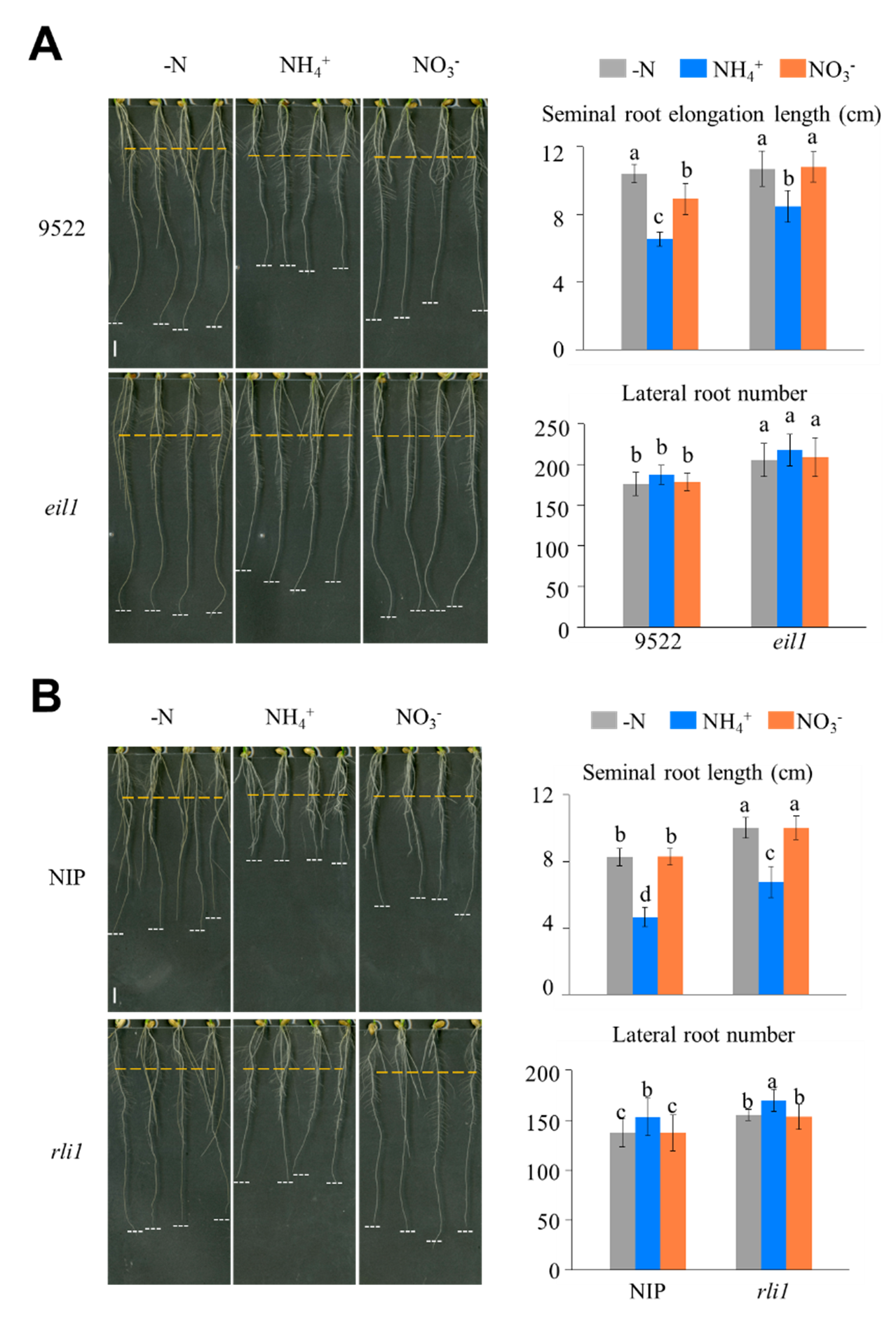

Supplement: Supplementary Figure 8 — Phenotypes under different nitrogen treatments of oseil1 mutants and osrli1 mutants. (A): Images of the oseil1 mutant and its 9522 background, with measurements of the seminal and lateral roots number. 9522 is the genetic background in which the oseil1 mutant has been constructed. (B): Images of the osrli1 mutant and its NIP background, with measurements of the seminal and lateral roots number. NIP is the genetic background in which the osrli1 mutant has been constructed. The orange dotted line indicates the position of the root tip when the seedlings were transferred to medium supplied with different N. The white dotted line indicates the position of the root tip when the seedlings were treated for 4 days. Different letters correspond to the post-hoc Tuckey’s test significance (p.value=0.05), performed after a two-way ANOVA test, and show significant differences between the samples. [file Image8.tif]

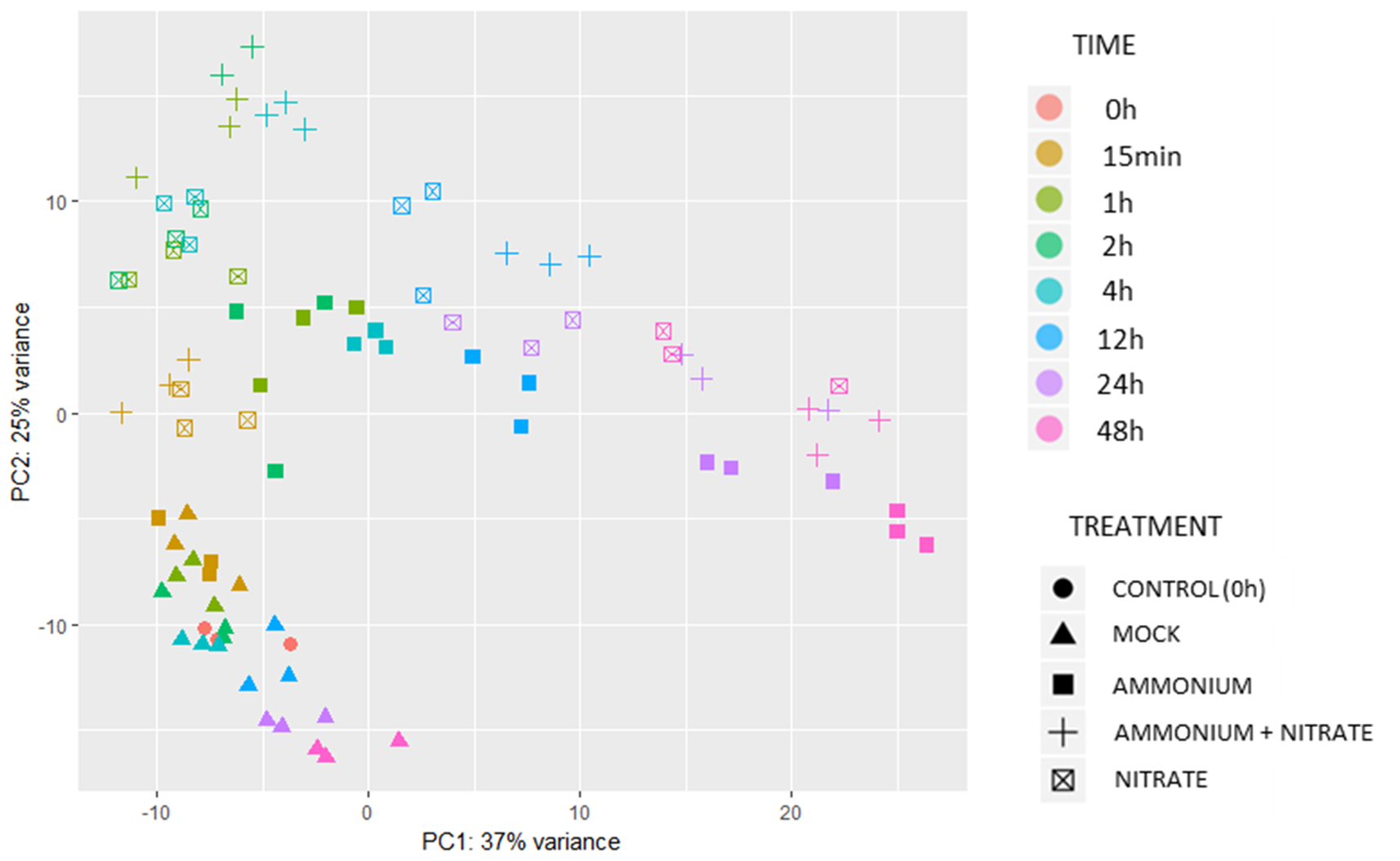

Supplement: Supplementary Figure 9 — Principal component analysis of the roots RNA-seq samples. Principal component analysis of the of the DESeq2 output normalized with the varianceStabilizingtransFormation() function in roots. [file Image9.tif]

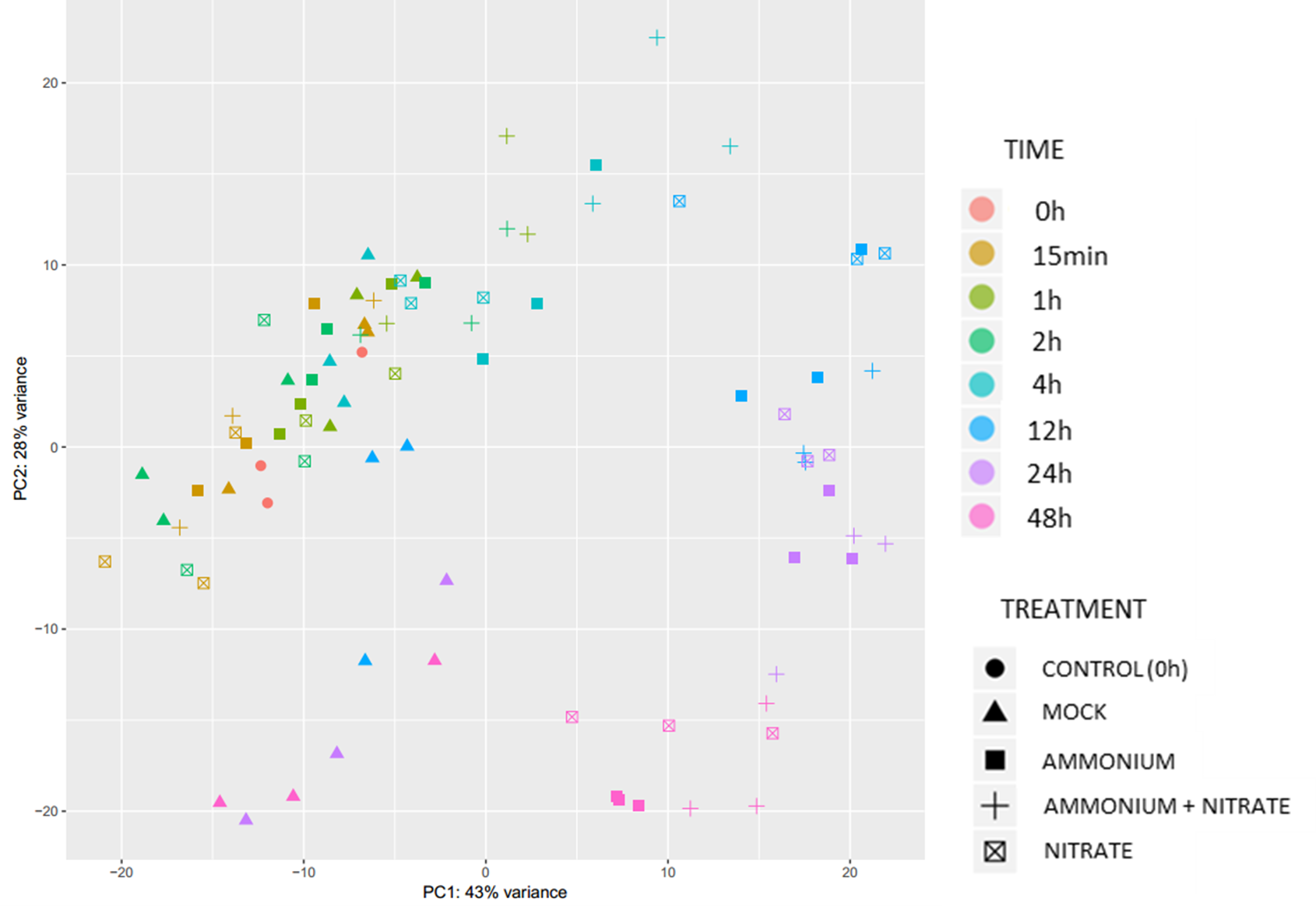

Supplement: Supplementary Figure 10 — Principal component analysis of the shoots RNA-seq samples. Principal component analysis of the of the DESeq2 output normalized with the varianceStabilizingTransformation() function in shoots. [file Image10.tif]
